# ETV4 mediates dosage-dependent prostate tumor initiation and cooperates with p53 loss to generate prostate cancer

**DOI:** 10.1126/sciadv.adc9446

**Published:** 2023-04-05

**Authors:** Dan Li, Yu Zhan, Naitao Wang, Fanying Tang, Cindy J. Lee, Gabriella Bayshtok, Amanda R. Moore, Elissa W. P. Wong, Mohini R. Pachai, Yuanyuan Xie, Jessica Sher, Jimmy L. Zhao, Makhzuna Khudoynazarova, Anuradha Gopalan, Joseph Chan, Ekta Khurana, Peter Shepherd, Nora M. Navone, Ping Chi, Yu Chen

**Affiliations:** ^1^ Human Oncology and Pathogenesis Program, Memorial Sloan Kettering Cancer Center, New York, NY 10065, USA.; ^2^ Sandra and Edward Meyer Cancer Center and Department of Physiology and Biophysics, Weill Cornell Medicine, New York, NY 10065, USA.; ^3^ Weill Cornell Graduate School of Medical Sciences, Weill Cornell Medicine, New York, NY 10065, USA.; ^4^ Department of Medical Oncology, Memorial Sloan Kettering Cancer Center, New York, NY 10065, USA.; ^5^ Department of Pathology, Memorial Sloan Kettering Cancer Center, New York, NY 10065, USA.; ^6^ Department of Medicine, Thoracic Oncology Service, Memorial Sloan Kettering Cancer Center, New York, NY 10065, USA.; ^7^ Institute for Computational Biomedicine, Weill Cornell Medical College, New York, NY 10021, USA.; ^8^ Department of Physiology and Biophysics, Weill Cornell Medical College, New York, NY 10021, USA.; ^9^ Englander Institute for Precision Medicine, Weill Cornell Medicine, New York, NY 10021, USA.; ^10^ Genitourinary Medical Oncology, MD Anderson Cancer Center, 1515 Holcombe Boulevard, Houston, TX 77030, USA.; ^11^ Department of Medicine, Division of Hematology and Medical Oncology, Weill Cornell Medicine, New York, NY 10065, USA.; ^12^ Department of Medicine, Memorial Sloan Kettering Cancer Center, New York, NY 10065, USA.

## Abstract

The mechanisms underlying *ETS*-driven prostate cancer initiation and progression remain poorly understood due to a lack of model systems that recapitulate this phenotype. We generated a genetically engineered mouse with prostate-specific expression of the *ETS* factor, ETV4, at lower and higher protein dosage through mutation of its degron. Lower-level expression of ETV4 caused mild luminal cell expansion without histologic abnormalities, and higher-level expression of stabilized ETV4 caused prostatic intraepithelial neoplasia (mPIN) with 100% penetrance within 1 week. Tumor progression was limited by p53-mediated senescence and *Trp53* deletion cooperated with stabilized ETV4. The neoplastic cells expressed differentiation markers such as Nkx3.1 recapitulating luminal gene expression features of untreated human prostate cancer. Single-cell and bulk RNA sequencing showed that stabilized ETV4 induced a previously unidentified luminal-derived expression cluster with signatures of cell cycle, senescence, and epithelial-to-mesenchymal transition. These data suggest that *ETS* overexpression alone, at sufficient dosage, can initiate prostate neoplasia.

## Teaser

Sufficient protein dosage of ETV4 can directly transform prostate epithelial cells, which recapitulates early human disease.

## INTRODUCTION

Prostate cancer is the most common malignancy and the second leading cause of cancer deaths in the United States, with an estimated 268,490 new cases and 34,500 deaths being anticipated during 2022 (*1*). Genetic rearrangements that lead to transcriptional up-regulation of *ETS* family transcription factors including *ERG* and three highly conserved polyomavirus enhancer activator 3 (PEA3) subfamily members—*ETV1*, *ETV4*, and *ETV5—*are prevalent and mutually exclusive in prostate cancer; they occur early in the natural history of the disease (*2*–*6*). Oncogenomic analysis indicates that *ETS* rearrangements define one genetic subtype of prostate cancer that is most common in Caucasian men; *FOXA1* mutations define a second subtype that is most common in Asian men; and *SPOP* mutations define a third subclass that represents ~10% in each of Caucasian, African American, and Asian men (*7*, *8*).

Despite intense interest, the mechanisms by which *ETS* overexpression leads to prostate cancer tumorigenesis and disease progression are still not well understood, largely owing to the lack of model systems that show robust tumor initiation. Several genetically engineered mouse (GEM) models that overexpress *ERG* or *ETV1* showed either minimal or no phenotype early time points (*9*–*14*), thus failing to recapitulate the cancer initiating ability of ETS translocation implicated by human genetics. In the setting of *Pten* deletion that by itself robustly mediates neoplasia in the mouse prostate, ERG and ETV1 overexpression reproducibly promotes cancer progression in numerous studies (*9*, *15*–*17*). However, in human prostate cancer, while *PTEN* deletion and *ETS* rearrangement are highly correlated, *PTEN* deletion is less common, is less clonal, and can be heterogenous in *ETS*-rearranged cancers, suggesting that *ETS* rearrangement occurs earlier in human prostate tumorigenesis (*18*, *19*). *TP53* alterations also significantly correlate with *ETS* rearrangement in primary prostate cancer (*3*, *20*), but this interaction has not been explored in mouse models.

The lack of robust oncogenic phenotype in exiting GEM models of *ETS* overexpression may be due to relatively insufficient protein expression of *ETS* transcription factors achieved in previous *ETS*-driven murine prostate cancer models compared to that in human *ETS*-positive prostate cancers (*16*, *21*) and/or tumor cell-intrinsic barriers that prevent tumor progression. In one transgenic line of higher ERG overexpression, there was focal murine prostatic intraepithelial neoplasia (mPIN) at 6 months of age and sporadic invasive cancer at 2 years of age (*10*, *14*). However, the phenotype was still delayed and sporadic with low penetrance.

The protein dosage of PEA3 subfamily of *ETS* transcription factors ETV1, ETV4, and ETV5 is regulated through both transcription and ubiquitylation-mediated protein degradation by E3 ubiquitin ligase COP1, a tumor suppressor in both human prostate cancer and in GEM models of prostate cancer (*22*–*24*). In the prostate TCGA cohort, aberrations of highly homologous *ETV1* and *ETV4* genes comprised 4.8 and 3.3% of patients, respectively, and we chose *ETV4* for modeling. Here, we have developed conditional murine models of wild-type ETV4 (ETV4^WT^) and stabilized ETV4 (V_70_P_71_D_72_-AAA and V_78_P_79_D_80_-AAA, ETV4^AAA^), harboring mutations at the COP1-binding sites and therefore resistant to COP1-mediated degradation. We demonstrate that the stabilized ETV4^AAA^ significantly increased the protein dosage of ETV4. ETV4^AAA^ expression alone initiates widespread mPIN throughout the prostate epithelium within 2 weeks of activation. The mPIN fails to further progress to invasive cancer due to ETV4-mediated simultaneous activation of the p53-dependent senescence program, and ETV4^AAA^ cooperates with *Trp53* loss to promote the development of focally invasive prostate cancer. The neoplastic cells retain expression of prostate lineage differentiation markers such as Nkx3.1 similar to most cases of localized human prostate cancer. In contrast, *Pten* loss–driven murine prostate cancers generally lose Nkx3.1 and are de novo castration resistant and distinct in human primary adenocarcinoma (*25*–*28*). The *ETV4^AAA^; Trp53^LoxP/LoxP^* model maintains luminal gene expression features, which are common features of human prostate cancer but not captured well in other mouse models.

## RESULTS

### Expression of stabilized ETV4 is sufficient to induce prostate neoplasia

Previous studies showed that transgenic and conditional *Rosa26*-mediated *ETS* overexpression in mice did not approximate the high expression level in human prostate cancer, which may account for the minimal phenotypes observed in these models. To examine the role of *ETS* transcription factor protein dosage in prostate cancer initiation, we took advantage of the degron containing two adjacent COP1-binding sites shared by PEA3 subfamily of *ETS* transcription factors—*ETV1*, *ETV4*, and *ETV5* (*22*–*24*, *29*, *30*). We chose to model ETV4 as a representative PEA3 factor and mutated both COP1 degrons from ExxVPD to ExxAAA (ETV4^AAA^). We expressed enhanced green fluorescent protein (EGFP) vector control, ETV4^WT^, and ETV4^AAA^ in A375 melanoma cells and found that, at baseline, protein level of ETV4^AAA^ was significantly higher than that of ETV4^WT^. Treatment with MG132 that inhibits proteosomal degradation increased ETV4^WT^ protein levels and endogenous ETV4 in vector-infected control cells but only minimally affected ETV4^AAA^ protein level, indicating that ETV4^AAA^ is constitutively stable (fig. S1A). To evaluate the possibility that, beyond protein stability, the mutations introduced in ETV4^AAA^ may alter its transcriptional function, we expressed EGFP, ETV4^WT^, and ETV4^AAA^ in two prostate cancer cell lines, 22Rv1 and PC3, and performed RNA sequencing (RNA-seq) analysis. In 22Rv1 cells, the protein level of ETV4^AAA^ was significantly higher than that of ETV4^WT^ due to degradation by COP1. PC3 cells harbor a deletion in COP1 and have increased baseline protein levels of ETV4 (*22*, *31*). In PC3 cells, exogenously expressed ETV4^AAA^ and ETV4^WT^ exhibited similar protein levels (fig. S1B). Principal components analysis (PCA) of RNA-seq showed that the first principal component (PC) was separated by cell line and that the second PC was separated by ETV4 expression. In 22RV1 cells, ETV4^AAA^ caused a much greater expression change as ETV4^WT^ in the same direction. In PC3 cells that have baseline higher levels of ETV4, the transcriptome perturbation was less in general, and ETV4^WT^ caused a greater change in gene expression (fig. S1C). We compared the global transcriptome change induced by ETV4^AAA^ and ETV4^WT^. In 22Rv1 cells, there was a high correlation [Pearson correlation coefficient (*r*) = 0.72], and ETV4^AAA^ induced a greater change (fig. S1D). In PC3 cells, the global transcriptome change was also strongly correlated (Pearson *r* = 0.76), and ETV4^WT^ induced slightly higher gene expression changes. These data suggest that ETV4^AAA^ regulates a similar transcriptome to ETV4^WT^.

**Fig. 1.**
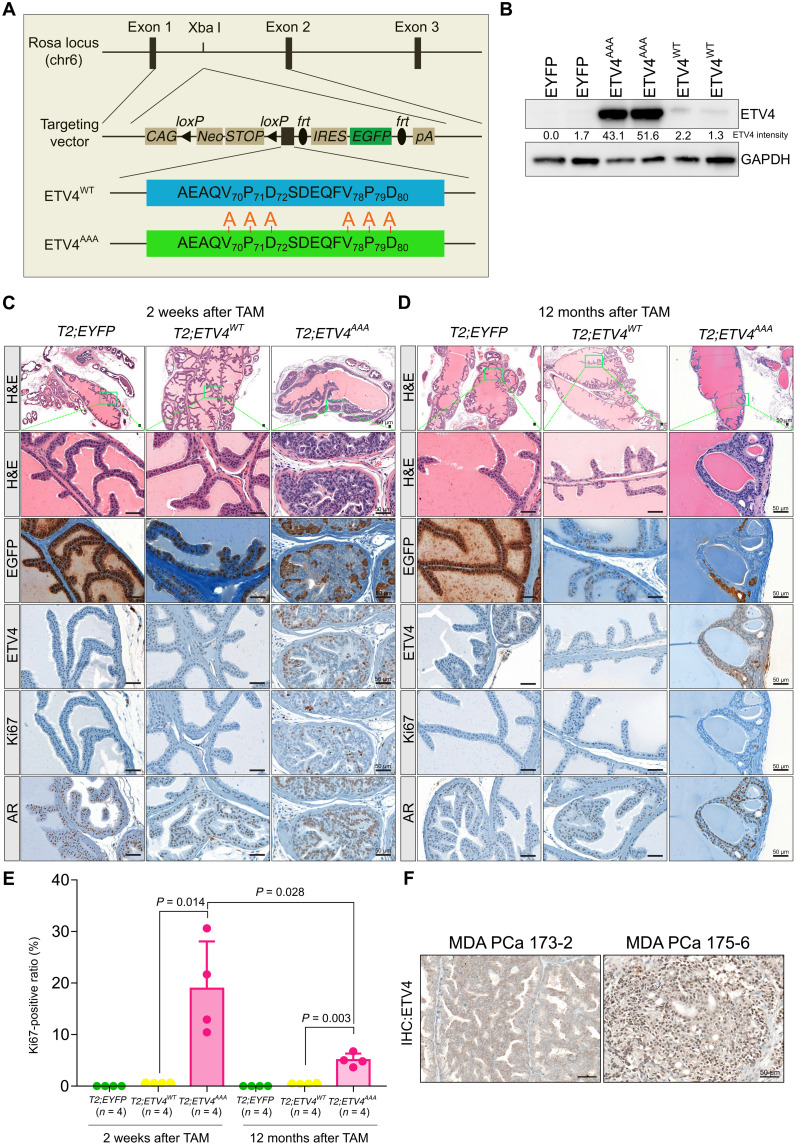
Expression of stabilized ETV4 (ETV4^AAA^) induces prevalent mPin. (**A**) Schematic for generating mice with transgenic expression of human *ETV4^WT^* and *ETV4^AAA^* driven by the chicken actin CMV composite promoter (CAG) knocked into the mouse *Rosa26* locus. The targeting plasmid was electrophoresed into albino C57BL/6J embryonic stem cells to generate the *Rosa26-CAG-LSL-ETV4^WT^* and *Rosa26-CAG-LSL-ETV4^AAA^* mice. (**B**) ETV4 protein expression in mouse prostate from two mice of each genotype quantified by Western blot 1 week after tamoxifen (TAM) treatment. The intensity of ETV4 bands is quantified using ImageJ. GAPDH, glyceraldehyde-3-phosphate dehydrogenase. (**C** and **D**) Two weeks (C) and 12 months (D) after TAM treatment, histological analysis anterior prostate of *T2; EYFP*, *T2; ETV4^WT^*, and *T2; ETV4^AAA^* mice prostate is shown by hematoxylin and eosin (H&E). The expression of EGFP, ETV4, AR, and Ki67 are analyzed with immunohistochemistry (IHC) staining. Scale bars, 50 μm. (**E**) The Ki67-positive luminal cell ratios are quantified on IHC stained slides. (**F**) IHC staining of ETV4 on MDA PCa patient-derived xenograft (PDX) samples. Error bars are SD.

We generated mouse models with conditional expression of *ETV4^WT^* and *ETV4^AAA^* followed by *IRES-EGFP* driven by the CAG promoter knocked into the *Rosa26* locus (Fig. 1A). For prostate luminal cell–specific and temporally controlled expression, we crossed these two *ETV4* alleles, as well as CAG-driven conditional *EYFP* allele, with our previously described *Tmprss2-CreER^T2^* (*T2*) mice (*32*–*35*). We used intraperitoneal injection of tamoxifen (TAM) to initiate transgene expression in prostate luminal cells in 6- to 8-week-old *T2; EYFP*, *T2; ETV4^WT^*, and *T2; ETV4^AAA^* male mice. One week after TAM injection, Western blot analysis of prostate lysates showed that ETV4 protein expression was higher in *T2; ETV4^AAA^* mice compared to that in *T2; ETV4^WT^* mice, consistent with the increased stability of ETV4^AAA^ protein in the murine prostate (Fig. 1B). We next examined the histology of the prostate 2 weeks after TAM administration. By then, there are EGFP- or enhanced yellow fluorescent protein (EYFP)–positive cells in all prostate lobes of all three genotypes, indicating that the *Tmprss2-CreER^T2^* was active. At this early time point, *ETV4^AAA^*-expressing mice already developed prevalent prostatic intraepithelial neoplasia (mPIN) with enlarged nuclei, prominent nucleoli, and cribriform growth in the anterior and dorsal prostate (Fig. 1C and fig. S2). The mPIN retained robust androgen receptor (AR) immunostaining and exhibited increased proliferation marked by Ki67 staining. In contrast, the prostates from *ETV4^WT^*-expressing mice were indistinguishable from those expressing EYFP. While GFP immunohistochemistry (IHC) showed prevalent EGFP- or EYFP-positive cells in murine prostates of all three genotypes, ETV4 IHC detected weak nuclear ETV4 protein in ETV4^WT^ prostate but strong nuclear ETV4 expression in the *ETV4^AAA^*-expressing prostate cells (Fig. 1C). Despite the expression of ETV4^AAA^ marked by positive EGFP and ETV4 IHC, we did not observe mPIN in the lateral and ventral lobes of the ETV4^AAA^ prostate (fig. S2). This highlights a lobe-specific sensitivity to specific oncogenic transformation as observed in other genetic engineered models of prostate cancer (*25*, *36*).

To determine whether high ETV4 protein expression alone is sufficient to drive progression to invasive cancer, we administered TAM in 6- to 8-week-old mice and aged them for 6, 9, and 12 months for phenotypic assessment. Unexpectedly, the mPIN of the anterior and dorsal prostate of *T2; ETV4^AAA^* mice regressed over time, and only residual areas of EGFP-positive mPIN were observed at 6, 9, and 12 months after TAM injection (Fig. 1D and fig. S3, A to C). Ki67 staining revealed that these residual areas with *ETV4^AAA^* expression continue to exhibit increased proliferation. However, the Ki67-positive luminal cell ratio was significantly reduced in ETV4^AAA^ mice at 12 months compared with that at 2 weeks after TAM administration ETV4^AAA^ (Fig. 1E). The prostates of *T2; EYFP* and *T2; ETV4^WT^* mice did not exhibit neoplastic phenotype over the entire 12-month period of observation (fig. S3C).

To determine whether the protein expression level of ETV4^AAA^ in the mouse anterior prostate is comparable to that in human prostate cancer, we performed ETV4 IHC using the same conditions on a patient-derived xenograft (PDX) model with TMPRSS2-ETV4 fusion (MDA PCa 175-6) and a control PDX (MDA PCa 173-2) from MD Anderson (MDA PCa program) deposited in biobank (*37*). We observed a strong nuclear staining in MDA PCa 175-6, suggesting that ETV4^WT^ protein expression is lower than that in human prostate cancer and that ETV4^AAA^ is clinically relevant (Fig. 1F).

We chose *Tmprss2-CreER^T2^* to follow luminal cells over time after a single Cre-mediated induction event. We further compared the induction with *Probasin-Cre* (*Pbsn-Cre*), the most commonly used Cre driver in the prostate epithelium, that mediates continued recombination in prostate epithelial cells from puberty (*38*) to generate the *Pbsn-Cre*; *ETV4^AAA^* mice. We found that, at 6, 9, and 12 months of age, approximately 50% of the *Pbsn-Cre; ETV4^AAA^* mice exhibited mPIN phenotype in anterior lobe and dorsal lobe (fig. S3, D and E). The areas of mPIN were marked by positive EGFP staining. We reasoned that the continuous induction of ETV4^AAA^ though a nonconditional Cre driver results in relatively constant phenotype over time. Despite the continuous induction that maintained mPIN throughout the time studied, there was no evidence of disease progression over time. These data suggest that the expression of *ETV4^AAA^* can directly induce mPIN with little latency and that persistent mPIN phenotype requires maintenance of the *ETV4^AAA^* expression in prostate epithelial cells; however, *ETV4^AAA^* expression alone is insufficient to mediate progression to invasive cancer.

### Stabilized ETV4 induces widespread changes in chromatin landscape to mediate tumorigenesis and senescence

We next examined the molecular impact of stabilized ETV4 in the unique phenotypes associated with prostate tumorigenesis. Recent data have shown that *ETS* translocation alters the chromatin enhancer landscape and the AR cistrome in prostate cancer (*9*, *39*–*41*). To characterize ETV4^AAA^-induced effects on open chromatin landscape and gene expression, we performed assay for transposase-accessible chromatin using sequencing (ATAC-seq) and RNA-seq, respectively. We chose the 2-week time point to enrich for direct targets. To specifically analyze prostate epithelial cells with the recombined allele, we sorted EPCAM-positive and EYFP- or EGFP-positive prostate epithelial cells by fluorescence-activated cell sorting (FACS) from *T2; EYFP*, *T2; ETV4^WT^*, and *T2; ETV4^AAA^* murine prostates (fig. S4A).

**Fig. 2.**
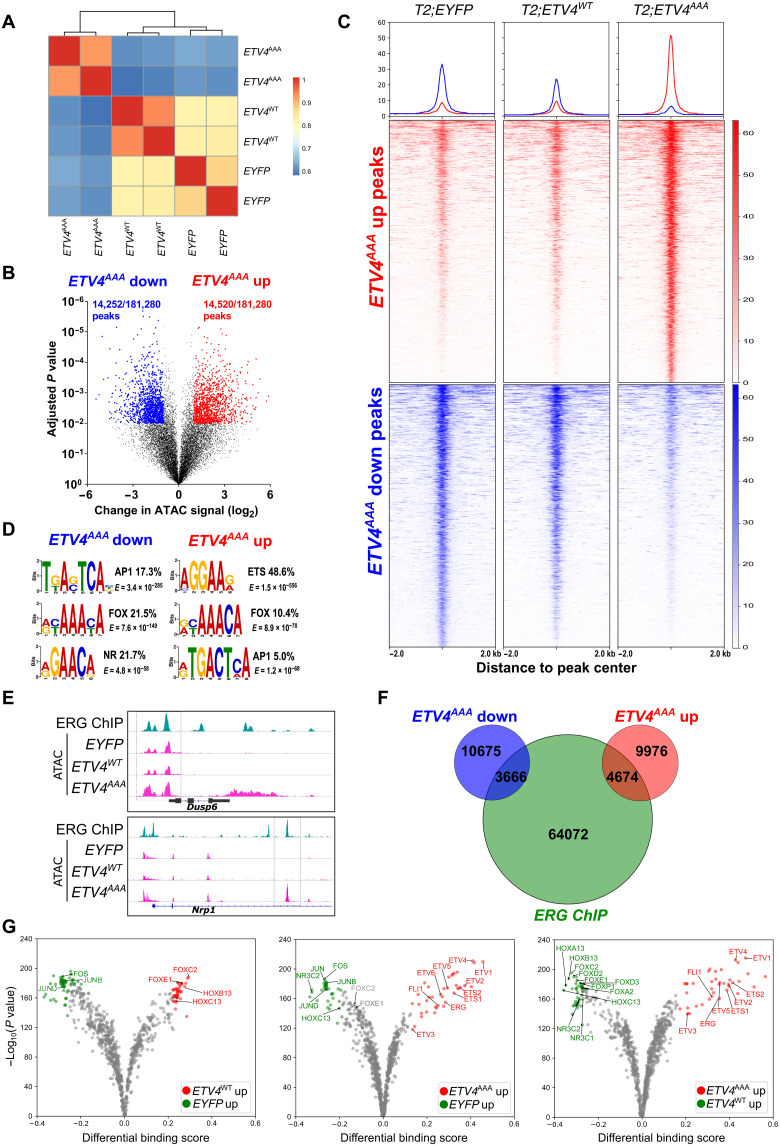
ETV4^AAA^ induces widespread changes in chromatin landscape. (**A**) Clustering of ATAC signal intensity at mapped peaks of EYFP-, ETV4^WT^-, and ETV4^AAA^-expressing cells. (**B**) Volcano plot of ATAC peaks, with significantly increased and decreased peak of ETV4^AAA^-expressing cells compared with EYFP-expressing cells show [adjusted *P* < 0.01, fold change (FC) > 2]. (**C**) Peaks with increased and decreased signal of ETV4^AAA^-expressing cells compared with that of EYFP-expressing cells are shown with heatmap. (**D**) The enriched motifs of ETV4^AAA^–up-regulated or ETV4^AAA^–down-regulated ATAC peaks are shown. (**E**) The chromatin accessibility of well-characterized ETS targets, *Dusp6* and *Nrp1* from ERG chip-seq data of *Pb-Cre4*; *Rosa26*^*ERG*/*ERG*^ mice and ATAC-seq data of EYFP-, ETV4^WT^-, and ETV4^AAA^-expressing cells are shown. (**F**) Venn plot shows the overlap between ERG-binding sites from ERG ChIP-seq and significantly increased and decreased ATAC peaks of ETV4^AAA^-expressing cells compared with that of EYFP-expressing cells. (**G**) Volcano plot of footprinting motif analysis on ATAC-seq data shows the increased binding of transcription factors of EYFP-, ETV4^WT^-, and ETV4^AAA^-expressing cells.

ATAC-seq profiles of FACS-sorted cells exhibited expected insert size distribution of open chromatin and nucleosome peaks enriched around known promoters (fig. S4, B and C). Unsupervised clustering of ATAC signal intensity showed that *ETV4^WT^*- and *EYFP*-expressing cells clustered closely, while *ETV4^AAA^*-expressing cells exhibited marked changes in the accessible chromatin landscape (Fig. 2A). We found that ~15% of all ATAC peak loci exhibited changes in peak intensity between *EYFP*- and *ETV4^AAA^*-expressing cells [adjusted *P* < 0.01, fold change (FC) > 2], with approximately equal number of peaks with significantly increased and decreased ATAC signals (Fig. 2B). Among these peaks that are changed by ETV4^AAA^, we found that ETV4^WT^ expression induced changes in ATAC-seq signal in the same direction as that of ETV4^AAA^ expression, but with much smaller magnitude (Fig. 2C), indicating ETV4 dosage-dependent changes in global chromatin accessibility. Motif analysis using MEME–chromatin immunoprecipitation (ChIP) revealed that the *ETS* motif was the most strongly enriched motif among ETV4^AAA^–up-regulated ATAC peaks, occurring at 48.6% of these loci (table S1). The FOX and AP1 motifs were significantly enriched at both ETV4^AAA^–up-regulated and ETV4^AAA^–down-regulated peaks, and the nuclear receptor motif was enriched at the down-regulated peaks (Fig. 2D). Consistently, well-characterized *ETS* targets such as *Dusp6* and *Nrp1* showed increased chromatin accessibility by ETV4^AAA^ expression at previously defined ERG-binding sites in murine prostates with exogenous ERG expression (Fig. 2E) (*9*, *27*). Globally, ERG-binding sites overlap with 31% of ETV4^AAA^–up-regulated ATAC-seq sites and 25% of ETV4^AAA^–up-regulated ATAC-seq sites (Fig. 2F). ATAC-seq footprinting analysis of transcription factor binding sites that minimizes experimental bias showed significantly enhanced binding of ETS transcription factors in *ETV4^AAA^* compared not only with *EYFP*-positive but also with *ETV4^WT^*-positive cells (Fig. 2G and table S2), further indicating an ETV4 dosage-dependent impact on chromatin landscape.

**Fig. 3.**
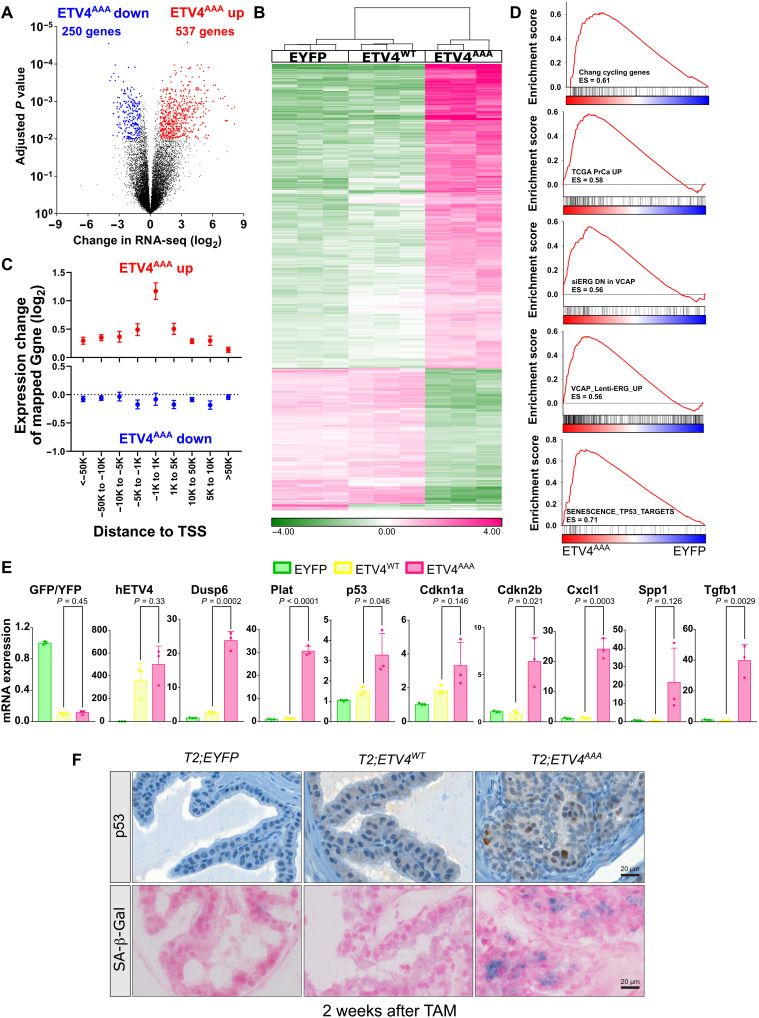
ETV4^AAA^ induces p53 and senescence. (**A**) Volcano plot of up-regulated genes and down-regulated genes in ETV4^AAA^-expressing cells compared with that in EYFP-expressing cells by RNA-seq. (**B**) Hierarchical clustering of genes significantly changed by ETV4^AAA^-expressing cells compared that by with EYFP- and ETV4^WT^-expressing cells. (**C**) The expression of genes mapped to increased (upper panel) or decreased peaks (lower panel) in ETV4^AAA^-expressing cells compared with that in EYFP-expressing cells is shown by integrative analysis of ATAC-seq and RNA-seq data. (**D**) The enriched gene sets in ETV4^AAA^-expressing cells compared with that in EYFP-expressing cells are analyzed using gene set enrichment analysis. (**E**) The expression of individual genes in EYFP, ETV4^WT^ and ETV4^AAA^ prostate cells are shown using RNA-seq data. *n* = 3 for each phenotype. (**F**) The expression of p53 in anterior prostate of EYFP, ETV4^WT^, and ETV4^AAA^ mice 2 weeks after TAM treatment is analyzed with IHC staining. The senescence-associated β-galactosidase (SA-β-Gal) is analyzed with SA-β-Gal staining. Scale bars, 20 μm. Error bars are SD.

We next analyzed the ETV4^AAA^-induced transcriptome changes. Compared to *EYFP*-expressing control, *ETV4^AAA^* expression induced a greater number of statistically up-regulated genes (537) than down-regulated genes (250) (adjusted *P* < 0.05, FC > 2) (Fig. 3A). Hierarchical clustering of these genes showed that ETV4^WT^ induced only minimal expression changes compared to EYFP control (Fig. 3B). Integrative analysis of ATAC-seq and RNA-seq showed that the closer a peak with increased ATAC signal is mapped to the transcriptional start site (TSS) of a gene, the greater its mean increase in expression; for example, peaks mapped within 1 kb of TSS exhibited a ~2.3-fold increase in expression. Genes mapped to decreased peaks exhibited only minimal decrease in expression (1.02-fold) (Fig. 3C). These data suggest that ETV4^AAA^ primarily serves as a dosage-dependent transcriptional activator and can enhance transcription through increasing chromatin accessibility at promoters.

To determine the gene expression programs activated by ETV4^AAA^ in prostate epithelial cells, we performed gene set enrichment analysis (GSEA) using ~3000 curated genes sets and custom prostate cancer gene sets. There were many more gene sets enriched among genes significantly up-regulated by ETV4^AAA^ expression than among genes down-regulated by ETV4^AAA^ expression, also suggesting that ETV4^AAA^ is primarily a transcriptional activator (fig. S4D). Among enriched gene sets are those genes associated with cell cycle and proliferation (CHANG_CYCLING_GENES), prostate cancer tumorigenesis in human prostate cancer (TCGA_PrCa_UP), and ERG target genes in prostate cancer (VCAP_siERG_DN and VCAP_Lenti_ERG_UP) (Fig. 3D). We further used GSEA to query gene sets generated from well-established prostate cancer GEM models, including sets of genes up-regulated and down-regulated of the Hi-MYC mouse compared to normal prostate (*36*), of the Pten-deleted mouse compared to normal prostate (*25*), and of ERG-positive versus ERG-negative prostate in the setting Pten deletion (*9*). We found that these sets were significantly enriched (fig. S4E). These data indicate that stabilized ETV4 expression induces an *ETS*-driven prostate oncogenesis program. Notably, gene sets associated with p53-induced senescence were also significantly and positively enriched with ETV4^AAA^ expression (Fig. 3D and table S3).

Examination of individual genes showed that transcript levels of ETV4–internal ribosomal entry site (IRES)–EGFP was similar between ETV4^WT^- and ETV4^AAA^-expressing prostate cells; canonical *ETS* transcriptional targets, e.g., *Dusp6* and *Plat*, were mildly induced by ETV4^WT^ and much more significantly induced by ETV4^AAA^ (Fig. 3E). p53 (*Trp53*), its downstream target p21 (*Cdkn1a*), and senescence-associated genes *Cdkn2b*, *Cxcl1*, *Spp1*, and *Tgfb1* were all significantly up-regulated in ETV4^AAA^-expressing prostate cells compared to that in EYFP and ETV4^WT^ controls. ETV4^AAA^ prostates stained positive for p53 by IHC and for senescence-associated β-galactosidase (Fig. 3F). These data suggest that high dosage of ETV4 expression is required to drive an ETS-mediated oncogenic transcriptional program but simultaneously induces *Trp53*- and p53-associated senescence, which likely limit prostate cancer development and progression from mPIN in ETV4^AAA^-expressing prostate.

### p53 loss cooperates with stabilized ETV4 to promote invasive prostate carcinoma

**Fig. 4.**
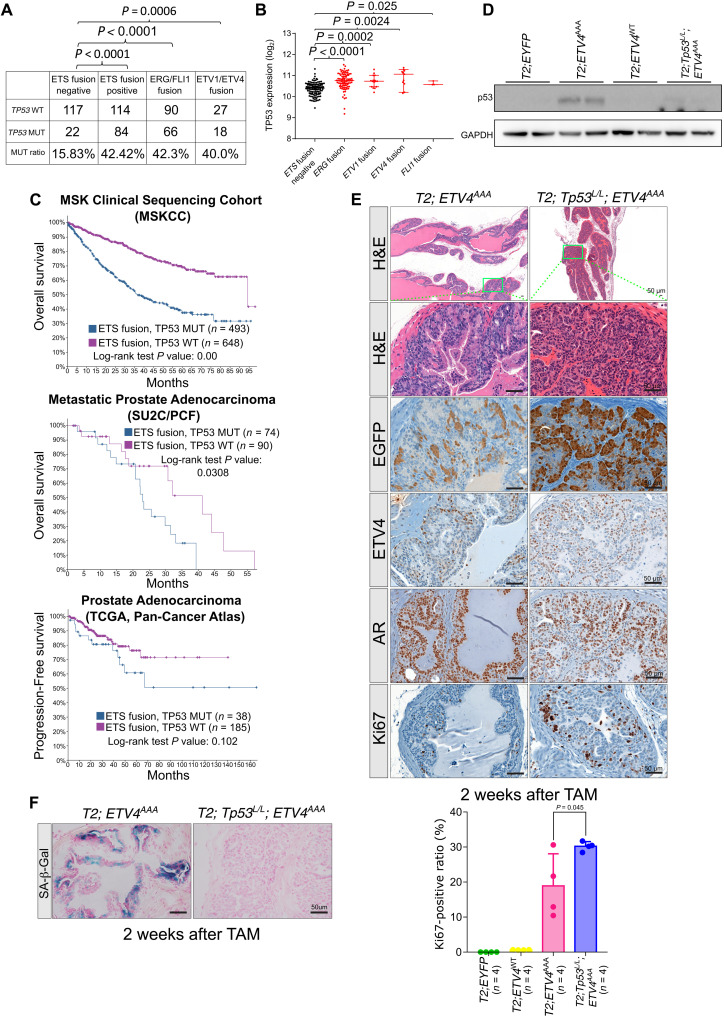
*P53* loss reduces ETV4^AAA^ induces senescence. (**A**) *TP53* alteration rate (mutation or copy number loss) in ETS fusion–positive samples and ETS fusion–negative samples from TCGA data of primary prostate cancer is quantified. (**B**) TP53 mRNA expression levels are analyzed in ETS fusion–positive samples compared with that in ETS fusion–negative samples among TP53 wild-type samples from TCGA data of primary prostate cancer. (**C**) The survival probability of patients with TP53 loss in ETS-positive prostate cancer. (**D**) The expression of p53 in EYFP, ETV4^WT^, ETV4^AAA^, and ETV4^AAA^;*Tp53*^L/L^ mice 2 weeks after TAM treatment are analyzed with Western blot. (**E**) Representative H&E (low magnification and high magnification of indicated area) and IHC of EGFP (antibody recognizes both EGFP and EYFP), AR, ETV4, and Ki67 of anterior prostate 2 weeks after TAM treatment in *T2; Tp53^L/L^; ETV4^AAA^* mice. The Ki67-positive luminal cell ratios are quantified on IHC stained slides. (**F**) Representative SA-β-Gal staining of anterior prostate 2 weeks after TAM treatment in *T2; ETV4^AAA^* and *T2; Tp53^L/L^; ETV4^AAA^* mice. Error bars are SD.

We analyzed the TCGA dataset of primary prostate cancer to correlate *TP53* genetic aberrations and expression with *ETS* translocation status (*3*). *TP53* genetic alteration rate (mutation or copy number loss) is significantly higher in *ETS* fusion–positive samples (42.42%) than that in *ETS* fusion–negative samples (15.83%), consistent with analysis of the Memorial Sloan Kettering Cancer Center cohort (Fig. 4A) (*20*). We reasoned that *ETS* overexpression may induce p53 expression that selects for p53 loss. Thus, we compared *TP53* RNA expression level between *ETS* fusion–positive samples and *ETS* fusion–negative samples specifically in *TP53* wild-type tumors and found *TP53* expression level to be significantly higher in *ETS* fusion–positive prostate cancer (Fig. 4B). To assess the clinical significance of TP53 loss in ETS-positive prostate cancer, we mined clinical outcome data in three datasets (Fig. 4C): (i) TCGA dataset of primary prostate cancer using endpoint of progression-free survival (*3*), (ii) MSKCC dataset of aggressive prostate cancer with endpoint of overall survival (*42*), and (iii) SU2C/PCF International Dream Team dataset of castrate-resistant prostate cancer (CRPC) with endpoint of overall survival (*43*, *44*). In each dataset, TP53 mutation conveyed a least a trend for poor outcome in ETS-positive prostate cancer (*P* value not signification for TCGA). These data indicate that aberrant high level of *ETS* expression activates p53 and downstream senescence programs and that p53 loss might be a cooperative event that overcomes the barrier for human prostate tumorigenesis and progression.

To study the role of p53 in the ETV4^AAA^-mediated senescence and tumor progression, we crossed in the *Trp53^LoxP/LoxP^* mice into the *T2; ETV4^AAA^* mice to generate *T2; Trp53^LoxP/LoxP^; ETV4^AAA^* mice. Two weeks after TAM injection, Western blot of prostate lysates shows decreased p53 protein (Fig. 4D). *T2; Trp53^LoxP/LoxP^; ETV4^AAA^* mice exhibited more profound mPIN compared to *T2; ETV4^AAA^* mice with the lumen filled with epithelial cells positive for ETV4 and EGFP immunostaining and increased Ki67-positive luminal cell ratio compared to *T2; ETV4^AAA^* mice (Fig. 4E). Moreover, senescence-associated β-galactosidase staining was diminished in *T2; Trp53^LoxP/LoxP^; ETV4^AAA^* compared to that in *T2; ETV4^AAA^* prostates (Fig. 4F). These data suggest that the activation of Trp53 by ETV4^AAA^ constrains tumorigenesis.

### scRNA-seq shows that ETV4^AAA^ drives a new luminal cluster that will evolve into prostate neoplastic cells

**Fig. 5.**
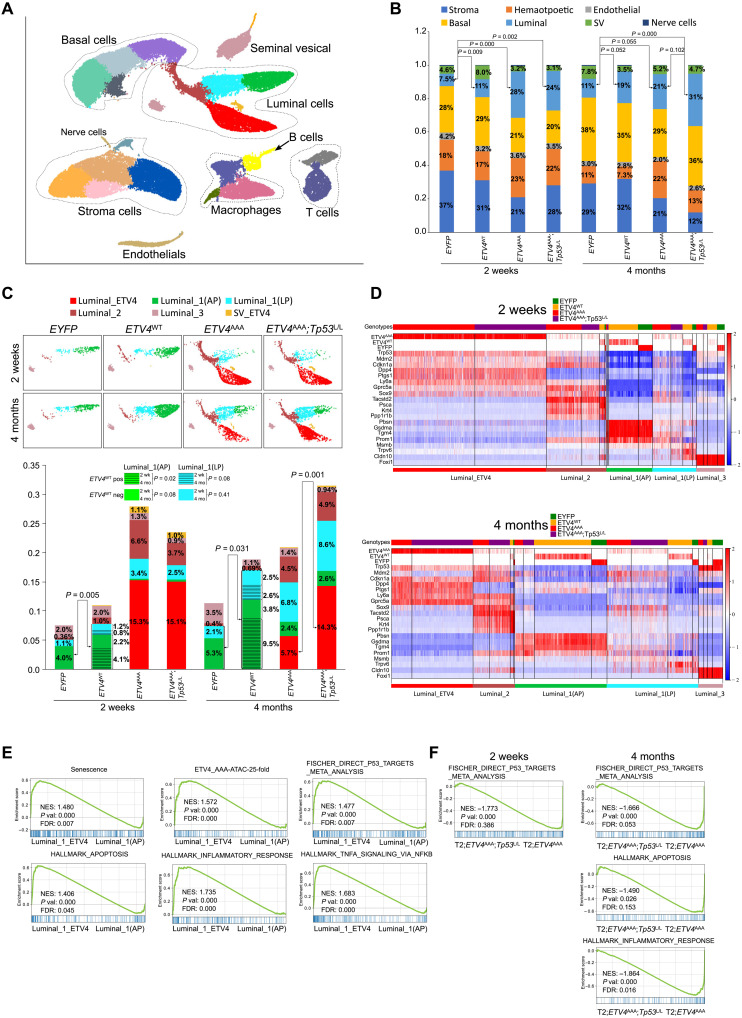
A single-cell atlas of the mPin induced by ETV4^AAA^. (**A**) Single-cell census of the prostates. Shown is uniform manifold approximation and projection (UMAP) of single-cell RNA-seq (scRNA-seq) profiles colored by Leiden clustering of 25 subsets and labeled post hoc. (**B**) Bar graph showing cell percentage of each lineage (three mice per sample; *P* values are estimated using *t* test). SV: seminal vesical. (**C**) Luminal subsets are shown separately for each sample, and cell percentage of each subset was quantified (three mice per sample; *P* is from *t* test). (**D**) Heatmap shows the highly differentially expressed genes (DEGs) for each Luminal clusters, 2 weeks and 4 months after TAM treatment. The top labels each cell by genotype of the mice. The gene expression matrix was normalized to counts per million (CPM) and log_2_(CPM + 1), and gene imputation was performed. The EYFP, ETV4^WT^, and ETV4^AAA^ expressions were shown with the raw data at the top three rows. ETV4^WT^ and ETV4^AAA^ were the same gene reads and separated based on the sample name. (**E**) The enriched gene sets in Luminal_1_ETV4 compared with that in Luminal_1(AP) are analyzed with GSEA. (**F**) The enriched gene sets in T2; *Tp53^L/L^; ETV4^AAA^* mice compared with that in T2; *ETV4^AAA^* mice, 2 weeks and 4 months after TAM treatment, of cells in Luminal_1_ETV4 cluster are analyzed with GSEA.

To explore the transcriptional heterogeneity of ETV4-expressing cells and to study the regulatory relationships between ETV4-positive cells and other cell populations in microenvironment, we performed single-cell RNA-seq (scRNA-seq) on FACS-sorted live cells from prostates of *T2; EYFP*, *T2; ETV4^WT^*, *T2; ETV4^AAA^*, and *T2; Trp53^LoxP/LoxP^; ETV4^AAA^* mice 2 weeks and 4 months after TAM treatment. We analyzed single transcriptomes from 48,926 single cells in eight groups of the four genotypes and two time points (*n* = 3 mice for each group), which include 19,854 genes, with a median of 1973 genes per cell and a median of 2039 cells per mouse. To visualize single cells of the global atlas, we used uniform manifold approximation and projection (UMAP) for dimension reduction. We then performed Leiden clustering (*45*) and identified 25 clusters, including six luminal epithelial clusters, four basal epithelial clusters, five fibroblast clusters, and six hematopoietic clusters (Fig. 5A and table S4).

Quantification of the cell numbers in each cluster showed that, in *T2; EYFP* wild-type mice, there was expansion of epithelial cells (both luminal and basal) over time from 2 weeks to 4 months (35% versus 49%, *P* = 0.003) (Fig. 5B and table S5). At both time points, *T2; ETV4^WT^* mice exhibited an increased percentage of luminal cells with similar percentage of basal cells compared to *T2; EYFP* mice (Fig. 5B, cyan), suggesting that *ETV4^WT^* can mediate luminal expansion. *T2; ETV4^AAA^* prostates exhibited more pronounced luminal expansion at 2 weeks but no further increase at 4 months, consistent with the early neoplastic phenotype observed by histology. In *T2; ETV4^AAA^;Trp53^LoxP/LoxP^* prostate, there was similar luminal expansion at 2 weeks and further increase at 4 months (Fig. 5B and table S5). These data are consistent with our observation that p53-mediated senescence limits the expansion and persistence of mPIN phenotype of *ETV4^AAA^*-expressing prostate cells (Fig. 1, C and D, and fig. S3, A and B).

We next analyzed the identities of luminal cell clusters by the marker gene expression. We then annotated the luminal clusters based on the highly expressed marker genes and quantified the cell numbers in each group (fig. S5A). We first focused on *T2; EYFP* wild-type mice and identified the common luminal cell populations: luminal 1 (L1), L2, and L3 that had been found in previous studies (fig. S5A and table S6) (*35*, *46*, *47*), adopting nomenclature from Karthaus *et al.* (*46*). The Luminal_1 cells are secretory cells that comprise the bulk of the distal prostate. We further separated them into two subclusters: Luminal_1(AP) that highly expresses anterior prostate marker genes *Pbsn*, *Gsdma*, and *Tgm4*; and Luminal_1(LP) that highly expresses lateral prostate marker genes *Msmb* and *Trpv6* (*47*). Luminal_2 cells are found in the proximal prostate and invaginated tips of distal prostate; express *Tacstd2* (Trop2), *Ly6a* (Sca1), *Psca*, and *Krt4*; and exhibit enhanced progenitor characteristics (*35*). Luminal_3 cells are ionocytes defined by expression of the transcription factor *Foxi1* (fig. S5A and table S6).

Compared to *T2; EYFP* wild-type prostates, *T2;ETV4^WT^* prostates did not comprise any new luminal clusters, suggesting that ETV4^WT^ cannot transform luminal cells. However, *T2;ETV4^WT^* prostates exhibited expansion of Luminal_1 clusters compared with *T2;EYFP* that is more pronounced from 2 weeks to 4 months (Fig. 5C and table S5; 5.1% versus 8.3%, *P* = 0.005, at 2 weeks; and 7.4% versus 18.4%, *P* = 0.03, at 4 months). Furthermore, within the *T2;ETV4^WT^* prostates, the expansion of Luminal_1 clusters over time was mostly due to *ETV4^WT^*-expressing cells (dashed green and dashed cyan). These data suggest that, while expression of *ETV4^WT^* did not cause detectable histologic phenotype or new gene expression cluster, its expression was able to cause luminal expansion over time at the single-cell level.

In contrast, expression of ETV4^AAA^ in *T2; ETV4^AAA^* and *T2; ETV4^AAA^;Trp53^LoxP/LoxP^* prostates generated two new luminal clusters that express *ETV4^AAA^* (Luminal_ETV4 and SV_ETV4) with distinct transcriptome features from previously described luminal clusters (Fig. 5C). The Luminal_ETV4 cells highly express L1 marker genes: CD26/Dpp4 and progenitor marker Sox9 (Fig. 5D). At 4 months after TAM administration, the percentage of Luminal_ETV4 cells in *T2; ETV4^AAA^;Trp53^LoxP/LoxP^* was significantly higher than that in *T2; ETV4^AAA^* mice, indicating that loss of p53 facilities the persistence of this neoplastic population and allowing for tumor progression over time (Fig. 5D). We next specifically analyzed expression of *Trp53* and downstream genes *Mdm2* and *Cdkn1a* (Fig. 5D and fig. S5, B and C). In control EYFP mice and in ETV4^WT^ mice, *Trp53*, *Mdm2*, and *Cdkn1a* were robustly expressed in Foxi1-positive Luminal_3 cells and lowly expressed in Luminal_1 cells. *Trp53* and downstream genes are up-regulated in the Luminal_ETV4 cluster compared to that in the Luminal_1_AP and Luminal_1_LP clusters, consistent with ETV4^AAA^-induced stress. Trp53^L/L^ mice exhibit a slightly decreased Trp53 expression likely due to nonsense mediated decay. Trp53 downstream genes including Mdm2 and Cdkn1a are also up-regulated in the Luminal-ETV4 cluster compared to that in Luminal_1 clusters. In Luminal-ETV4 cells, they are down-regulated after Trp53 deletion (Fig. 5D and fig. S5, B and C). The decrease in Mdm2 and Cdkn1a is more apparent in 4-month-old mice, possibly from selection of Trp53 completely lost cells.

We analyzed the altered signaling pathways in the Luminal_ETV4 cluster compared with that in Luminal_1(AP) using GSEA using GSEAPY package (Fig. 5E and table S7) (*48*). The senescence signaling, genes in the increased peaks of ATAC-seq in *ETV4^AAA^* mice, p53 signaling, apoptosis signaling, inflammatory signaling, and tumor necrosis factor–α signaling are significantly enriched in the Luminal_ETV4 cluster versus Luminal_1(AP) cluster (Fig. 5E and table S7). In Luminal_ETV4 cells of the *T2; Trp53^LoxP/LoxP^; ETV4^AAA^* prostate, the p53 signaling, apoptosis signaling, and inflammatory response pathways are significantly reduced in *Trp53^LoxP/LoxP^; ETV4^AAA^* mice versus *ETV4^AAA^* mice (Fig. 5F and tables S8 and S9).

In addition to ETV4^AAA^-expressing neoplastic luminal cells, *T2; ETV4^AAA^* and *T2; ETV4^AAA^;Trp53^LoxP/LoxP^* mice exhibited an increased percentage of Luminal_2 cells that did not express ETV4^AAA^ (Fig. 5, C and D). This suggests that ETV4^AAA^-expressing cells secrete paracrine signals that affect other populations. We compared the secretome of luminal populations. Luminal_ETV4 cells exhibit an altered secretome including *Tgfb1* (fig. S6A). Pathway analysis showed that extracellular matrix (ECM) organization and multiple growth factor pathways are up-regulated (fig. S6B). To verify the scRNA-seq results, we analyzed the gene expression of L2 marker gene Tacstd2/Trop2 using immunofluorescence staining (fig. S6C). While EGFP (indicating ETV4)–positive cells are Tacstd2 negative, there is a marked expansion of Tacstd2-positive cells in *ETV4^AAA^* mice and *Trp53^LoxP/LoxP^; ETV4^AAA^* mice. These Tacstd2-positive cells were found in regions of EGFP-positive, ETV4^AAA^-expressing cells, suggesting that ETV4^AAA^-positive cells, through paracrine interactions, expand the normal L2 population.

We next identified and quantified subclusters in basal, stroma, and hemopoietic populations. We identified four basal expression clusters. Basal_1 cells were found predominantly in *T2; EYFP* and *T2; ETV4^WT^* mice, Basal_2 cells were found exclusively in ETV4^AAA^-expressing mice, and Basal_3 cells had an intermediate gene expression and were found in all groups of mice (fig. S7A). Basal_2 cells highly express genes *Col17a1*, *Areg*, *Ly6d*, and *Cd44* (fig. S7B). We performed immunofluorescence staining of Col17a1 and found a high expression in Ck5-positive basal cells adjacent to EGFP-positive (ETV4^AAA^-expressing) luminal cells in ETV4^AAA^ mice (fig. S6D), suggesting that ETV4^AAA^ expression in the luminal cells induced paracrine gene expression changes in basal cells.

We identified five stroma clusters: Mesenchymal_1 to Mesenchymal_4 and myofibroblast (fig. S7C). Mesenchymal_1 is characterized by expression of *Lama2*, *Zeb1*, *Wnt2*, Wnt6, *Wnt10a*, and *Rorb*; Mesenchymal_2 is characterized by expression of *Sult1e1*, *Fgf10*, and *Rspo1*; Myofibroblast is characterized by expression of *Acta2*, *Myh11*, and *Notch3*. These clusters represent previously described Mesenchymal_1, Mesenchymal_2, and myofibroblast subsets (table S6) (*46*). We found a reduction in the percentage of all stroma clusters in 4-month *Trp53^LoxP/LoxP^; ETV4^AAA^* mice and further reduction of Wnt signaling–positive Mesenchymal_1 in *ETV4^AAA^* and *Trp53^LoxP/LoxP^; ETV4^AAA^* mice (fig. S7D). Mesenchymal_3 is characterized by expression of Sod3 and Osr1, which are mesenchymal stem cell markers (*49*, *50*). Mesenchymal_4 is characterized by expression of several ECM genes: Mgp, Bgn, and Col6a1 (fig. S7E), which are increased in *ETV4^AAA^* and *Trp53^LoxP/LoxP^; ETV4^AAA^* mice 2 weeks after TAM (fig. S7D).

The myeloid cells and lymphocytes are characterized using the expression of the marker genes (fig. S7F). Marker genes of M1 macrophages, M2 macrophages, and myeloid-derived suppressor cells (MDSCs) are adopted from other publications (*51*), and marker genes of cytotoxic T cells and regulatory T cells are shown in table S6. We found that there was no significant difference among the various M1, M2, and MDSCs in different genotypes, and they decreased in representation as the mice aged (fig. S7G).

### Stabilized ETV4^AAA^ cooperates with p53 loss to induce differentiated mouse invasive cancer

**Fig. 6.**
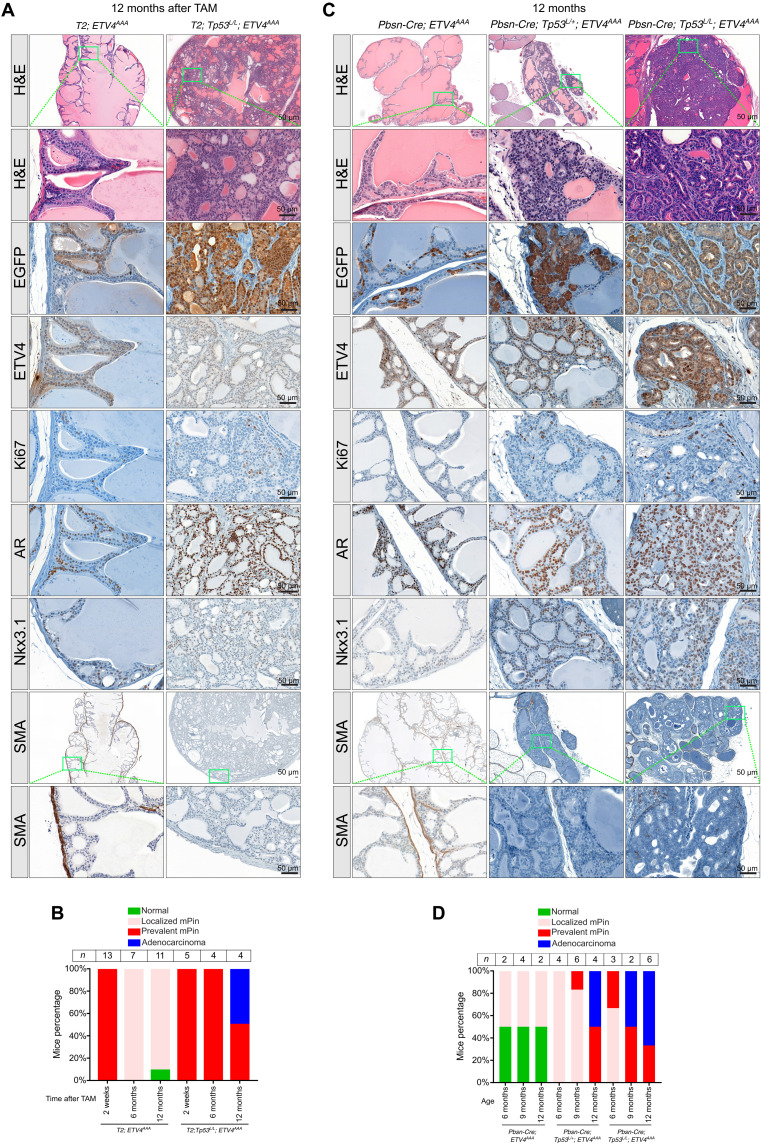
*P53* loss co-occurs with ETS fusion, and ETV4^AAA^ induces Nkx3.1-positive prostate cancer in the setting of p53 loss. (**A**) Representative H&E (low magnification and high magnification of indicated area) and IHC of GFP (antibody recognizes both EGFP and EYFP), ETV4, Ki67, AR, and Nkx3.1 in anterior prostate of 12-month-old *T2; Tp53^L/L^; ETV4^AAA^* mice. (**B**) The mice number of different phenotypes are quantified. (**C**) Histological changes of the anterior prostate of 12-month-old *Pbsn-Cre; Tp53^L/+^; ETV4^AAA^* and *Pbsn-Cre; Tp53^L/L^; ETV4^AAA^* mice are shown by H&E. The expression of EGFP, ETV4, Ki67, AR, Nkx3.1, and SMA is analyzed with IHC staining. Scale bars, 50 μm. (**D**) The mice number of different phenotypes are quantified.

We evaluated the long-term neoplastic phenotypes of the *T2;Trp53^LoxP/LoxP^; ETV4^AAA^* murine prostates 12 months after TAM administration. In contrast to the diminished neoplastic phenotype of the *T2*; *ETV4^AAA^*, we observed diffuse neoplasia with prevalent EGFP-positive cells, which maintained ETV4^AAA^, AR, and Nkx3.1 expression in the anterior prostate of the *T2; Trp53^LoxP/LoxP^; ETV4^AAA^* mice (Fig. 6, A and B), indicating that p53 loss is required in addition to high dosage level of ETV4 to drive prostate cancer progression. To further corroborate the effect of p53 loss on tumorigenesis in prostate cancer development and progression, we used a second murine model, the *Pbsn-Cre* model, and examined prostate tumorigenesis phenotypes of the *Pbsn-Cre; Trp53^LoxP/LoxP^; ETV4^AAA^* and *Pbsn-Cre; Trp53^LoxP/+^; ETV4^AAA^* GEM model with homozygous and heterozygous *Trp53* deletion at 6, 9, and 12 months of age. In both cohorts, the anterior prostates continued to exhibit diffuse neoplasia with prevalent EGFP- and ETV4^AAA^-positive cells at these later time points, which is different from that in *Pbsn-Cre; ETV4^AAA^* mice with intact p53 (Fig. 6, C and D, and fig. S8). Histological analysis of *ETV4^AAA^*; *Trp53^−/−^* anterior prostates driven by either T2-CreER^T2^ or Pbsn-Cre showed marked nuclear and nucleolar atypia, abnormal mitotic figures, and irregular infiltrative cribriform glands, which are typical features of human prostate cancer (Fig. 6, A and C). Loss of smooth muscle actin (SMA) is a marker of microinvasive disease in mouse models of prostate cancer (*25*, *36*). In *ETV4^AAA^*; *Trp53^−/−^* mice, we observed loss of SMA staining at 9 and 12 months of age, respectively (Fig. 6, A and C, and fig. S8). In contrast to other murine prostate cancer models (*25*, *36*, *52*), the tumor cells continued to maintain Nkx3.1 expression (Fig. 6, A and C), which is a pathologic hallmark for human prostate adenocarcinoma (*53*). These data suggest that *ETS* factors expressed at high dosage levels can initiate tumorigenesis, but it requires p53 loss to further promote the tumor progression and development of differentiated prostate cancer that accurately recapitulates human prostate cancer.

## DISCUSSION

Human genetic data have strongly implicated *ETS* gene rearrangements as an early cancer–initiating event in prostate cancer tumorigenesis, and IHC studies have shown that it can be detected in precancerous PIN (*2*–*5*, *19*, *54*). While *ETS* rearrangements significantly co-occur with *PTEN* and *TP53* deletion, IHC and genetics studies suggest that loss of these tumor suppressors may occur after *ETS* rearrangement (*19*, *20*, *42*).

We have previously shown that ERG reprograms the AR cistrome and, more recently, have demonstrated a direct interaction between ERG and AR and that ERG facilitates AR binding at specific *ETS*-AR motifs (*9*, *41*). Correspondingly, *ETS*-positive human prostate cancers exhibit a distinct enhancer landscape that enrich for ETS-, FOX-, and AR-binding sites (*40*). Studies in GEM models and in mouse prostate organoids have revealed that ERG overexpression and depletion of the *ETS* repressive tumor suppressor *ERF* in prostate epithelial cells enforce a luminal phenotype (*34*, *55*, *56*). While the luminal epithelial lineage is AR-dependent and thought to exhibit less plasticity, primary prostate cancers almost exclusively exhibit luminal phenotype and AR dependence. On the other hand, loss of tumor suppressors including *PTEN*, *TP53*, and *RB1* is correlated with dedifferentiation in human cancers and confers “lineage plasticity” in mouse models (*55*, *57*–*59*).

Despite these progresses, the mechanisms of *ETS*-mediated tumorigenesis and progression remain elusive. Models of prostate-specific *ETS* overexpression in GEM models do not cause early evidence of neoplasia (*9*–*14*). One potential explanation for the minimal phenotype is the low dosage level of achievable ETS (e.g., ERG or ETV1) proteins in previous *ETS*-driven murine prostate cancer models. Here, we show that the expression of *ETV4^WT^* only achieved low protein levels of ETV4, was weakly detectable by IHC, and caused no detectable histological changes. This was accompanied by mild gene expression changes and luminal expansion over time. Reasoning that the dosage of ETS protein is crucial in *ETS*-drive prostate cancer tumorigenesis, we created a low dosage (ETV4^WT^) level and a high dosage (ETV4^AAA^) level of ETV4 protein through increased ETV4 protein stability by mutating the conserved ExxVPD motifs responsible for COP1 degradation (*22*, *24*). We found that ETV4^AAA^ protein is stabilized, and the expression of ETV4^AAA^ significantly increases its downstream target gene expression in murine prostate cells compared with that of ETV4^WT^ (Fig. 3E). ETV4^AAA^ expression induces prevalent mPIN efficiently with no latency but simultaneously activates the p53-mediated senescence program that limits progression to prostate cancer. ETV4^AAA^ cooperates with p53 loss and promotes progression of mPIN to invasive prostatic adenocarcinoma. This cooperativity is in accord with human prostate cancer genomic data, where *TP53* alteration rate (mutation or copy number loss) is significantly higher in *ETS* fusion–positive samples than that in *ETS* fusion–negative samples (Fig. 4A) (*3*). Mechanistically, a high level of ETV4^AAA^ protein expression in prostate cells not only markedly alters the enhancer chromatin accessibility landscape, augmenting cell cycle progression, but also induces p53 and its downstream targets, including the cellular senescence programs, which underlines the molecular mechanism of mPIN regression in p53-wild-type and progression to prostate cancer with p53-loss in this murine prostate cancer model.

One unique feature of the ETV4^AAA^-driven murine prostate cancer model is that it maintains the characteristics of early-stage human prostate cancers, including prostate differentiation markers, such as Nkx3.1 expression. Comparably, prostates of *Pten* deletion, the most well-studied GEM model of prostate cancer, exhibit increased basal marker expression, lose Nkx3.1 expression, and are castration resistant de novo (*26*–*28*, *60*). The ETV4^AAA^-driven murine prostate cancer model establishes that ETS factor alone when expressed at high dosage can initiate tumorigenesis and cooperates with *TP53* loss for tumor progression.

## MATERIALS AND METHODS

### Generation of the Rosa26-CAG-LSL-ETV4^WT^ and Rosa26-CAG-LSL-ETV4^AAA^ mice

Rosa26 targeting was as described by Srinivas *et al.* (*61*) with modifications. We started the targeting vector with the CTV plasmid (Addgene, 15912; gift from K. Rajewsky), which contains the mouse *Rosa26* homology arm sequence from intron 1 of mouse *Rosa26* gene and *loxP-STOP-loxP* cassette followed by *frt*-*IRES*-*nlsEGFP*-*frt* cassette (*62*). We cloned human wild-type *ETV4* (NM_001986) and mutated *ETV4*^AAA^ cDNA into the plasmid in the middle of the two cassettes. The targeting plasmid was electrophoresed into albino C57BL/6J embryonic stem cells, and G418-resistant clones were isolated by the standard procedures. The clones were screened by Southern blotting. Positive clones were injected into C57BL/6J blastocysts by the MSKCC Mouse Genetics Core Facility, and chimeras were mated with albino C57BL/6J females. Germline transmission was confirmed in albino offspring using Southern blotting. For subsequent generations, genotyping was performed by quantitative polymerase chain reaction (PCR) using the following primers: hETV4_E3_F: GCCGCCCCTCGACTCTGAA; hETV4_E4_R: GAGCCACGTCTCCTGGAAGTGACT.

### DNA sequences of human wild-type *ETV4* (NM_001986) and mutated *ETV4* (*ETV4*^AAA^)

ATGGAGCGGAGGATGAAAGCCGGATACTTGGACCAGCAAGTGCCCTACACCTTCAGCAGCAAATCGCCCGGAAATGGGAGCTTGCGCGAAGCGCTGATCGGCCCGCTGGGGAAGCTCATGGACCCGGGCTCCCTGCCGCCCCTCGACTCTGAAGATCTCTTCCAGGATCTAAGTCACTTCCAGGAGACGTGGCTCGCTGAAGCTCAG**GTACCAGAC(GCAGCAGCC)**AGTGATGAGCAGTTT**GTTCCTGAT(GCTGCTGCT)**TTCCATTCAGAAAACCTAGCTTTCCACAGCCCCACCACCAGGATCAAGAAGGAGCCCCAGAGTCCCCGCACAGACCCGGCCCTGTCCTGCAGCAGGAAGCCGCCACTCCCCTACCACCATGGCGAGCAGTGCCTTTACTCCAGTGCCTATGACCCCCCCAGACAAATCGCCATCAAGTCCCCTGCCCCTGGTGCCCTTGGACAGTCGCCCCTACAGCCCTTTCCCCGGGCAGAGCAACGGAATTTCCTGAGATCCTCTGGCACCTCCCAGCCCCACCCTGGCCATGGGTACCTCGGGGAACATAGCTCCGTCTTCCAGCAGCCCCTGGACATTTGCCACTCCTTCACATCTCAGGGAGGGGGCCGGGAACCCCTCCCAGCCCCCTACCAACACCAGCTGTCGGAGCCCTGCCCACCCTATCCCCAGCAGAGCTTTAAGCAAGAATACCATGATCCCCTGTATGAACAGGCGGGCCAGCCAGCCGTGGACCAGGGTGGGGTCAATGGGCACAGGTACCCAGGGGCGGGGGTGGTGATCAAACAGGAACAGACGGACTTCGCCTACGACTCAGATGTCACCGGGTGCGCATCAATGTACCTCCACACAGAGGGCTTCTCTGGGCCCTCTCCAGGTGACGGGGCCATGGGCTATGGCTATGAGAAACCTCTGCGACCATTCCCAGATGATGTCTGCGTTGTCCCTGAGAAATTTGAAGGAGACATCAAGCAGGAAGGGGTCGGTGCATTTCGAGAGGGGCCGCCCTACCAGCGCCGGGGTGCCCTGCAGCTGTGGCAATTTCTGGTGGCCTTGCTGGATGACCCAACAAATGCCCATTTCATTGCCTGGACGGGCCGGGGAATGGAGTTCAAGCTCATTGAGCCTGAGGAGGTCGCCAGGCTCTGGGGCATCCAGAAGAACCGGCCAGCCATGAATTACGACAAGCTGAGCCGCTCGCTCCGATACTATTATGAGAAAGGCATCATGCAGAAGGTGGCTGGTGAGCGTTACGTGTACAAGTTTGTGTGTGAGCCCGAGGCCCTCTTCTCTTTGGCCTTCCCGGACAATCAGCGTCCAGCTCTCAAGGCTGAGTTTGACCGGCCTGTCAGTGAGGAGGACACAGTCCCTTTGTCCCACTTGGATGAGAGCCCCGCCTACCTCCCAGAGCTGGCTGGCCCCGCCCAGCCATTTGGCCCCAAGGGTGGCTACTCTTACTAG.

### Protein sequences of human wild-type ETV4 (NP_001977) and mutated ETV4 (ETV4^AAA^)

MERRMKAGYLDQQVPYTFSSKSPGNGSLREALIGPLGKLMDPGSLPPLDSEDLFQDLSHFQETWLAEAQ**VPD(AAA)**SDEQF**VPD(AAA)**FHSENLAFHSPTTRIKKEPQSPRTDPALSCSRKPPLPYHHGEQCLYSSAYDPPRQIAIKSPAPGALGQSPLQPFPRAEQRNFLRSSGTSQPHPGHGYLGEHSSVFQQPLDICHSFTSQGGGREPLPAPYQHQLSEPCPPYPQQSFKQEYHDPLYEQAGQPAVDQGGVNGHRYPGAGVVIKQEQTDFAYDSDVTGCASMYLHTEGFSGPSPGDGAMGYGYEKPLRPFPDDVCVVPEKFEGDIKQEGVGAFREGPPYQRRGALQLWQFLVALLDDPTNAHFIAWTGRGMEFKLIEPEEVARLWGIQKNRPAMNYDKLSRSLRYYYEKGIMQKVAGERYVYKFVCEPEALFSLAFPDNQRPALKAEFDRPVSEEDTVPLSHLDESPAYLPELAGPAQPFGPKGGYSY.

### Mouse alleles

All mouse studies are approved by MSKCC Institutional Animal Care and Use Committee under protocol 11-12-027. Institutional guidelines for the proper and humane use of animals in research were as follows. *Pb-Cre4* (*27*) and *Tmprss2-CreER^T2^* mice (*32*) were used as previously described. *ETV4*^WT^ and *ETV4*^AAA^ mice were crossed with *Tmprss2-CreER^T2^* or *Pb-Cre4* mice to generate *Tmprss2-CreER^T2^*, *ETV4*^WT^; *Tmprss2-CreER^T2^*, *ETV4*^AAA^; *Pb-Cre4*, and *ETV4*^WT^; *Pb-Cre4*, *ETV4*^AAA^ mice. Ai3(*B6.Cg-Gt(ROSA)26Sor^tm3(CAG-EYFP)Hze^/J*) mice of conditional CAG-driven EYFP expression were purchased from the Jackson Laboratory (*33*) and crossed with *Tmprss2-CreER^T2^* mice to generate the *Tmprss2-CreER^T2^*, *EYFP* mice as control. *Pten*^LoxP^ mice (*Pten*^tm2.1Ppp^) in which exons 4 and 5 are flanked by *LoxP* sites were used as previously described (*9*). *Trp53*^LoxP^ mice in which exons 2 to 10 are flanked by *LoxP* sites were used as previously described (*63*).

### Protein analysis

A375, 22Rv1, and PC3 cells were purchased from American Type Culture Collection (A375, CRL-1619; 22Rv1, CRL-2505; PC3, CRL-3471). For mouse prostates, all tissues were fixed at 4°C overnight in 4% paraformaldehyde. Tissue processing, embedding, sectioning, and IHC staining was performed. Antibodies for IHC and Western blotting are as follows: chicken anti-GFP (2 μg/ml for IHC; Abcam, ab13970), rabbit anti-AR [1:100 for IHC; Abcam, ER179(2), ab108341], rabbit anti-ETV4 (1:200 for IHC; 1:1,000 for Western blotting; Proteintech, 10684-1-AP), rabbit anti-Ki67 (1:200 for IHC; Abcam, ab16667), rabbit anti-SMA (0.4 μg/ml for IHC; Abcam, ab5694), rabbit anti-p53 (1:100 for IHC; 1:1000 for Western blotting; Novocastra, P53-CM5P-L), rabbit anti-Nkx3.1 (1:100 for IHC; AthenaES, 314), mouse anti–β-actin (1:50,000 for Western blotting; Sigma-Aldrich, A1978), and mouse anti–glyceraldehyde-3-phosphate dehydrogenase (1:10,000 for Western blotting; Santa Cruz Biotechnology, sc-59540). Tissue paraffin embedding, sectioning, and hematoxylin and eosin (H&E) staining were performed by the MSKCC core facility. IHC was performed by the MSKCC molecular cytology core using a Ventana Discovery XT. To generate lysates for Western blotting, tissue was homologized in radioimmunoprecipitation assay buffer using the FastPrep-24 system with Lysing Matrix A (MP Biomedicals).

Immunofluorescence staining of Tacstd2/Trop2 (1:100 dilution; R&D Systems, AF1122), Col17a1 (1:100 dilution; Abcam, ab184996), and Ck5 (1:100 dilution; BioLegend, no. 905501) was performed on frozen sections. Sections were permeabilized with 0.5% Triton X-100 for 10 min and blocked with 10% normal donkey serum for 30 min at room temperature. Primary antibodies were incubated overnight. Secondary antibodies with Alexa Fluor 647 and Alexa Fluor 555 (Thermo Fisher Scientific) conjugation were applied on the second day. Image were taken with Leica TCS SP5 upright confocal microscope.

### Mouse prostate digestion

Intraperitoneal injection of TAM was administered in 8-week-old mouse. Two weeks after TAM treatment, mouse prostate was digested 1 hour with collagenase/hyaluronidase (STEMCELL Technologies, no. 07912) and then 30 min with TrypLE Express Enzyme (Thermo Fisher Scientific, no. 12605028) at 37°C to isolate single prostate cells. The prostate cells were stained with phycoerythrin/cyanine 7–conjugated anti-mouse CD326 (EPCAM) antibody (BioLegend, 118216), and then, CD326 and EYFP double-positive cells were sorted out by flow cytometry, which are luminal cells mainly from anterior prostate and dorsal prostate. The mRNA or genomic DNA was extracted from these double-positive cells and then was used for ATAC-seq and RNA-seq analysis.

### ATAC-seq and primary data processing

ATAC-seq was performed as previously described (*64*). Primary data processing and peak calling were performed using ENCODE ATAC-seq pipeline (https://github.com/kundajelab/atac_dnase_pipelines). Briefly, paired-end reads were trimmed, filtered, and aligned against mm9 using Bowtie2 (*65*). PCR duplicates and reads mapped to mitochondrial chromosome or repeated regions were removed. Mapped reads were shifted +4/−5 to correct for the Tn5 transposase insertion. Peak calling was performed using Model-based Analysis of ChIP-seq (MACS2) (*66*), with *P* < 0.01 as cutoff. Reproducible peaks from two biological replicates were defined as peaks overlapped by more than 50%. On average, 25 million uniquely mapped pairs of reads were remained after filtering. The distribution of inserted fragment length shows typical nucleosome banding pattern, and the TSS enrichment score (reads that are enriched around TSS against background) ranges between 28 and 33, suggesting that the libraries have a high quality and were able to capture the majority of regions of interests.

### Differential peak accessibility

Reads aligned to peak regions were counted using R package GenomicAlignments_v1.12.2 (*67*). Read count normalization and differential accessible peaks were called with DESeq2_v1.16.1 in R 3.4.1 (*68*). Differential peaks were defined as peaks with adjusted *P* < 0.01 and |log_2_(FC)| > 2. For visualization, coverage bigwig files were generated using bamCoverage command from deepTools2 and were normalized using the size factor generated by DESeq2. The differential ATAC-seq peak density plot was generated with deepTools2 (*69*), using regions that were significantly more or less accessible in ETV4^AAA^ samples relative to EYFP samples.

### Motif analysis

Enriched motif was performed using MEME-ChIP 5.0.0 (*70*) with differentially accessible regions in ETV4^AAA^ relative to EYFP. ATAC-seq footprinting was performed using TOBIAS (*71*). First, ACACCorrect was run to correct Tn5 bias, followed by ScoreBigwig to calculate footprint score and, lastly, BindDetect to generate differential footprint across regions.

### RNA-seq analysis

The extracted RNA was processed for RNA-seq by the Integrated Genomics Core Facility at MSKCC. The libraries were sequenced on an Illumina HiSeq-2500 platform with 51–base pair (bp) paired-end reads to obtain a minimum yield of 40 million reads per sample. The sequenced data were aligned using STAR v2.3 (*72*) with GRCm38.p6 as annotation. DESeq2_v1.16.1 (*68*) was subsequently applied on read counts for normalization and the identification of differentially expressed genes (DEGs) between ETV4^AAA^ and EYFP groups, with adjusted *P* < 0.05 as threshold. Genes were ranked by sign[log_2_(FC)] × [−log(*P* value)] as input for GSEA analysis using “Run GSEA Pre-ranked” with 1000 permutations (*48*). The custom gene sets used in GSEA analysis are shown in table S2.

### Unsupervised hierarchical clustering

To get an overall sample clustering as part of quality control , hierarchical clustering was performed using pheatmap_v1.0.10 package in R on normalized ATAC-seq or RNA-seq data. It was done using all peaks or all genes, with Spearman or Pearson correlation as the distance metric. To have an overview of the differential gene expression from the RNA-seq data, unsupervised clustering was also performed on matrix with all samples as columns and scaled normalized read counts of DEGs between ETV4^AAA^ and EYFP as rows.

### Integrative analysis of ATAC-seq, RNA-seq, and ChIP-seq data

ERG ChIP-seq peaks were called using MACS 2.1 (*66*), with an FDR cutoff of *q* < 10^−3^ and the removal of peaks mapped to blacklist regions. Reproducible peaks between two biological replicates were identified as ETV4^AAA^ ATAC-seq peaks. ERG ChIP-seq peaks and ETV4^AAA^ ATAC-seq peaks were considered as overlap if peak summits were within 250 bp. To determine whether the overlap was significant, enrichment analysis was done using regioneR_v1.8.1 (*73*) in R, which counted the number of overlapped peaks between a set of randomly selected regions in the genome (excluding blacklist regions) and the ERG-ChIP seq peaks or ETV4^AAA^ ATAC-seq peaks. A null distribution was formed using 1000 permutation tests to compute the *P* value and *z* score of the original evaluation.

To assign ATAC-seq peaks to genes, ChIPseeker_v1.12.1 (*74*) in R was used. Each peak was unambiguously assigned to one gene with a TSS or 3′ end closest to that peak. Differential gene expression between ETV4^AAA^ and EYFP was evaluated using log_2_(FC) calculated by DESeq2. *P* values were estimated using Wilcoxon rank *t* test and Student’s *t* test.

### scRNA sequencing

*Tmprss2-CreER^T2^*, *EYFP*; *Tmprss2-CreER^T2^*, *ETV4*^WT^; *Tmprss2-CreER^T2^*, *ETV4*^AAA^; and *Tmprss2-CreER^T2^*, *ETV4*^AAA^; *Trp53*^L/L^ mice were euthanized 2 weeks or 4 months after TAM treatment (*n* = 3 mice for each genotype and time point). After euthanasia, the prostates were dissected out and minced with scalpel and then processed for 1 hour of digestion with collagenase/hyaluronidase (STEMCELL Technologies, no. 07912) and 30 min of digestion with TrypLE (Gibco, no. 12605010). Live single prostate cells were sorted out by flow cytometry as DAPI^−^. For each mouse, 5000 cells were directly processed with 10x Genomics Chromium Single Cell 3′ GEM, Library & Gel Bead Kit v3, according to the manufacturer’s specifications. For each sample, 200 million reads were acquired on NovaSeq platform S4 flow cell.

Reads obtained from the 10x Genomics scRNAseq platform were mapped to mouse genome (mm9) using the Cell Ranger package (10x Genomics). True cells are distinguished from empty droplets using scCB2 package (*75*). The levels of mitochondrial reads and numbers of unique molecular identifiers were similar among the samples, which indicates that there were no systematic biases in the libraries from mice with different genotypes. Cells were removed if they expressed fewer than 600 unique genes, less than 1500 total counts, more than 50,000 total counts, or greater than 20% mitochondrial reads. Genes detected in less than 10 cells and all mitochondrial genes were removed for subsequent analyses. Putative doublets were removed using the DoubletDetection package (*76*). The average gene detection in each cell type was similar among the samples. Combining samples in the entire cohort yielded a filtered count matrix of 48,926 cells by 19,854 genes, with a median of 6944 counts and a median of 1973 genes per cell and a median of 2039 cells per sample. The count matrix was then normalized to counts per million and log_2_(*X* + 1)–transformed for analysis of the combined dataset. The top 1000 highly variable genes were found using SCANPY (version 1.6.1) (*77*). PCA was performed on the 1000 most variable genes with the top 50 PCs retained with 29% variance explained.

To visualize single cells of the global atlas, we used UMAPs (https://arxiv.org/abs/1802.03426). We then performed Leiden clustering (*45*). Marker genes for each cluster were found with scanpy.tl.rank_genes_groups. Cell types were determined using the SCSA package, an automatic tool, based on a score annotation model combining DEGs and confidence levels of cell markers from both known and user-defined information (*78*). Heatmaps were performed for single cells based on log-normalized and scaled expression values of marker genes curated from literature or identified as highly differentially expressed.

DEGs between different clusters were found using MAST package (*79*), which were shown in heatmap. The logFC of MAST output was used for the ranked gene list in GSEA analysis (*48*). The custom gene sets used in GSEA analysis are shown in table S2.

Gene imputation was performed using MAGIC (Markov affinity-based graph imputation of cells) package (*80*), and imputated gene expression was used in the heatmap.

### Analysis of public human gene expression datasets

To analyze *TP53* RNA expression in human prostate cancer samples, we obtained normalized RNA-seq data from prostate cancer TCGA (www.firebrowse.org) (*3*). To assess the role of TP53 loss on progression-free survival and overall survival of ETS-positive prostate cancer, we used cBioportal (*81*). We queried MSKCC (queried on 12 October 2022), TCGA, and Stand-Up-To-Cancer cohorts; we selected patient with fusions of ERG, ETV1, ETV4, FL1, or TMPRSS2 and compared the overall survival between TP53 wild-type and TP53 mutant subsets (*3*, *42*, *44*).

### Statistics

All statistical comparisons between two groups were performed by Graphpad Prism 7.0 software used a two-tailed unpaired *t* test.

### Study approval

Mice were raised under the recommendation of MSKCC veterinary services in accordance to the MSKCC Institutional Animal Care and Use Committee (IACUC 11-12-029).

## Notes

View/request a protocol for this paper from *Bio-protocol*.
