# Supplementary material for: ETV4 mediates dosage-dependent prostate tumor initiation and cooperates with p53 loss to generate prostate cancer
Source: Sci Adv. 2023 Apr 5;9(14):eadc9446. doi: 10.1126/sciadv.adc9446 (PMC10075989; doi:10.1126/sciadv.adc9446)
Supplement: Supplementary file 1 — Figs. S1 to S8 Legends for tables S1 to S9 [file sciadv.adc9446_sm.pdf]

Science Advances
AAAS

Supplementary Materials for

ETV4 mediates dosage-dependent prostate tumor initiation and cooperates with p53 loss to generate prostate cancer

Dan Li *et al.*

Corresponding author: Yu Chen, cheny1@mskcc.org; Ping Chi, chip@mskcc.org

Sci. Adv. **9**, eadc9446 (2023)
DOI: 10.1126/sciadv.adc9446

The PDF file includes:

Figs. S1 to S8
Legends for tables S1 to S9

Other Supplementary Material for this manuscript includes the following:

Tables S1 to S9

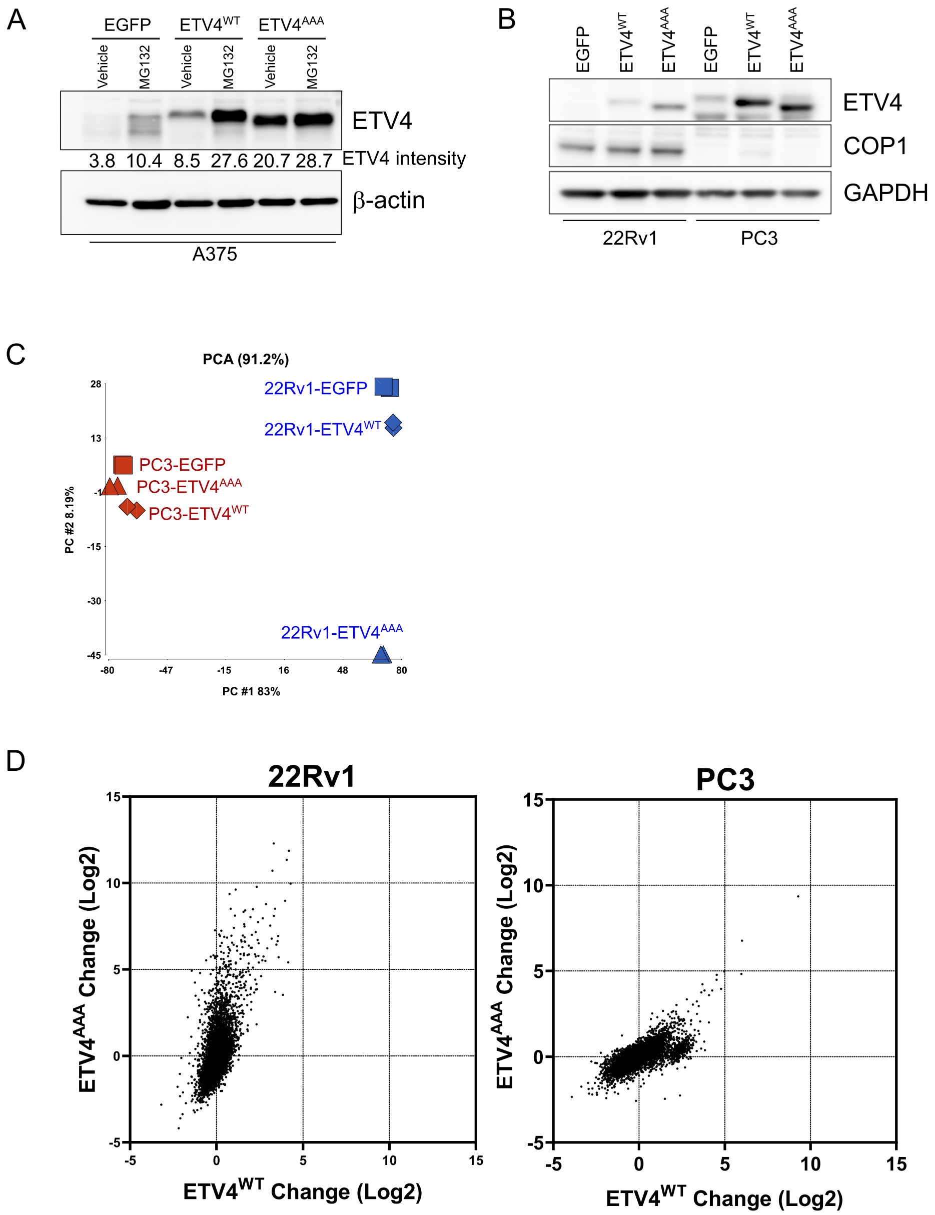
A
EGFP
ETV4WT
ETV4AAA
Vehicle
MG132
ETV4
3.8 10.4 8.5 27.6 20.7 28.7
ETV4 intensity
β-actin
A375
B
EGFP
ETV4WT
ETV4AAA
ETV4
COP1
GAPDH
22Rv1
PC3
C
PCA (91.2%)
22Rv1-EGFP
22Rv1-ETV4WT
PC3-EGFP
PC3-ETV4AAA
PC3-ETV4WT
22Rv1-ETV4AAA
PC #2 8.19%
PC #1 83%
D
22Rv1
PC3
ETV4AAA Change (Log2)
ETV4WT Change (Log2)

Figure S1

Fig. S1. ETV4^{AAA} shows increased stability and transcription activity *in vitro*

(A) Western blot of ETV4 expressed in A375 melanoma cells which are transduced with an pMSCV-IRES-EGFP vector expressing control EGFP, wild type human ETV4 (ETV4^{WT}) or ExxVPD to ExxAAA mutated ETV4 (ETV4^{AAA}), and treated with or without MG132 for 8 hours. The intensity of ETV4 bands is quantified using ImageJ. (B) Western blot of ETV4 and COP1 expressed in 22Rv1 and PC3 prostate cells which are transduced with an pMSCV-IRES-EGFP vector expressing control EGFP, ETV4^{WT} or ETV4^{AAA}. (C) PCA analysis of RNA-seq data of COP1 intact 22Rv1 and COP1-loss PC3 prostate cells which are transduced with an pMSCV-IRES-EGFP vector expressing control EGFP, ETV4^{WT} or ETV4^{AAA}. (D) Differentially expressed genes of ETV4^{AAA} compared with ETV4^{WT} in 22Rv1 and PC3 cells.

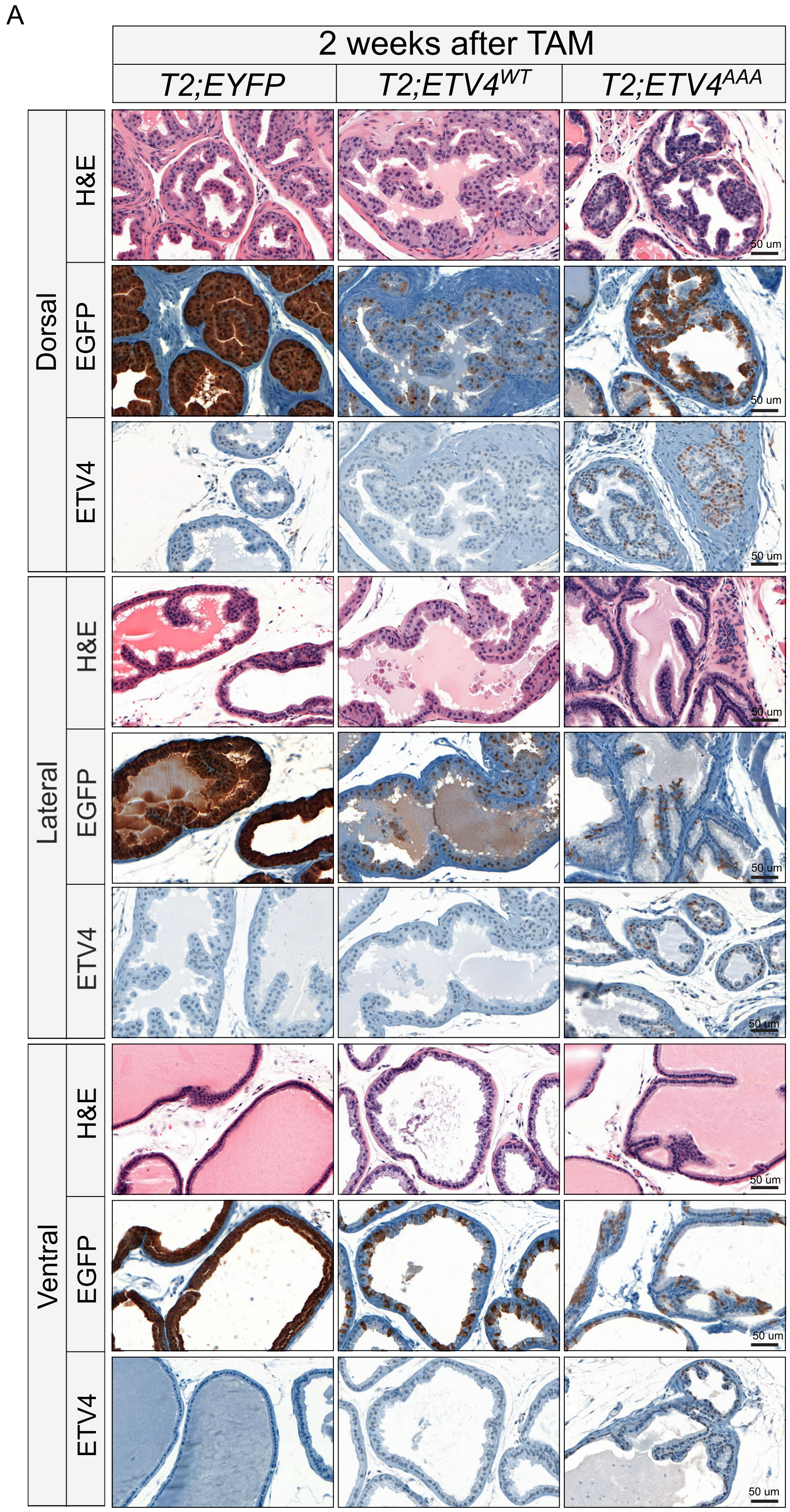
A
2 weeks after TAM
T2;EYFP
T2;ETV4WT
T2;ETV4AAA
Dorsal
Lateral
Ventral
H&E
EGFP
ETV4
50 um

Figure S2

Fig. S2. Expression of Stabilized ETV4 (ETV4^{AAA}) induces prevalent mPin in dorsal prostate

(A) 2 weeks after tamoxifen treatment, histological changes in dorsal, lateral and ventral prostates in *T2; EYFP*, *T2; ETV4^{WT}* and *T2; ETV4^{AAA}* mice are shown by H&E. The expression of EGFP and ETV4 is analyzed with IHC staining. Scale bar 50 um.

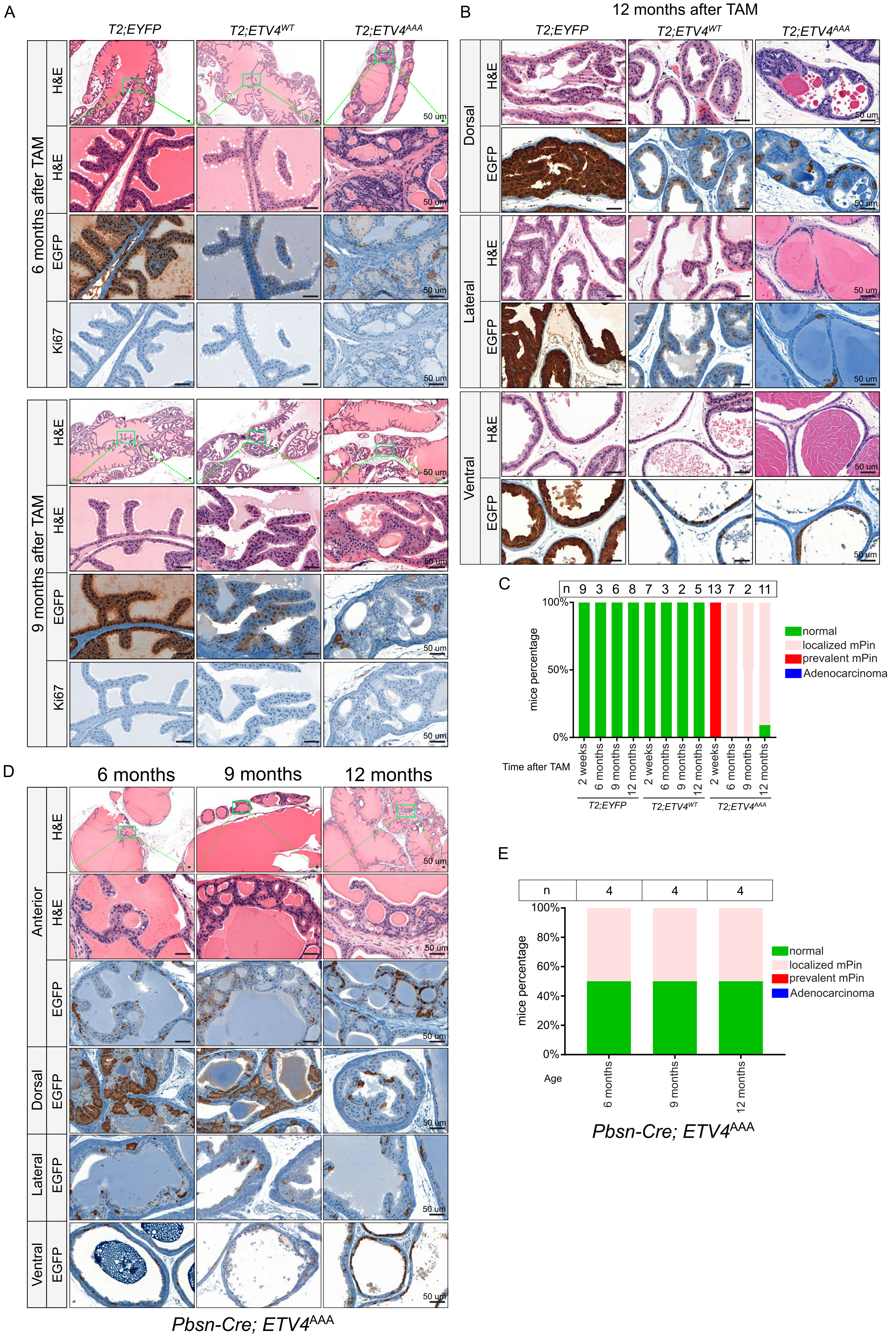

A
T2;EYFP
T2;ETV4^WT
T2;ETV4^AAA
6 months after TAM
9 months after TAM
H&E
EGFP
Ki67
50 um
B
12 months after TAM
Dorsal
Lateral
Ventral
C
n 9 3 6 8 7 3 2 5 13 7 2 11
100%
50%
0%
mice percentage
Time after TAM
2 weeks
6 months
9 months
12 months
normal
localized mPin
prevalent mPin
Adenocarcinoma
D
6 months
9 months
12 months
Anterior
Pbsn-Cre; ETV4^AAA
E
n 4 4 4
80%
60%
40%
20%
Age

Figure S3

Fig. S3. Prevalent mPin regresses to localized mPin in aged ETV4^{AAA} mice

(A) Representative H&E (low magnification and high magnification of indicated area) and IHC of GFP (antibody recognizes both EGFP and EYFP) of anterior prostate 6 and 9 months after tamoxifen treatment in *T2; EYFP, T2; ETV4^{WT}* and *T2; ETV4^{AAA}* mice. (B) 12 months after tamoxifen treatment, histological changes in dorsal, lateral and ventral prostates in *T2; EYFP, T2; ETV4^{WT}* and *T2; ETV4^{AAA}* mice are shown by H&E. The expression of EGFP is analyzed with IHC staining. (C) The mice number of different genotypes and phenotypes are quantified. Scale bar 50 um. (D) Representative H&E (low magnification and high magnification of indicated area) and IHC of GFP (antibody recognizes both EGFP and EYFP) of dorsal, lateral and ventral prostate 6-month, 9-month and 12-month-old *Pbsn-Cre; ETV4^{AAA}* mice. (E) Quantification of mouse prostate histology categorized as normal, localized mPIN, prevalent mPIN and adenocarcinoma in 6-month, 9-month and 12-month-old *Pbsn-Cre; ETV4^{AAA}* mice. Scale bar 50 um.

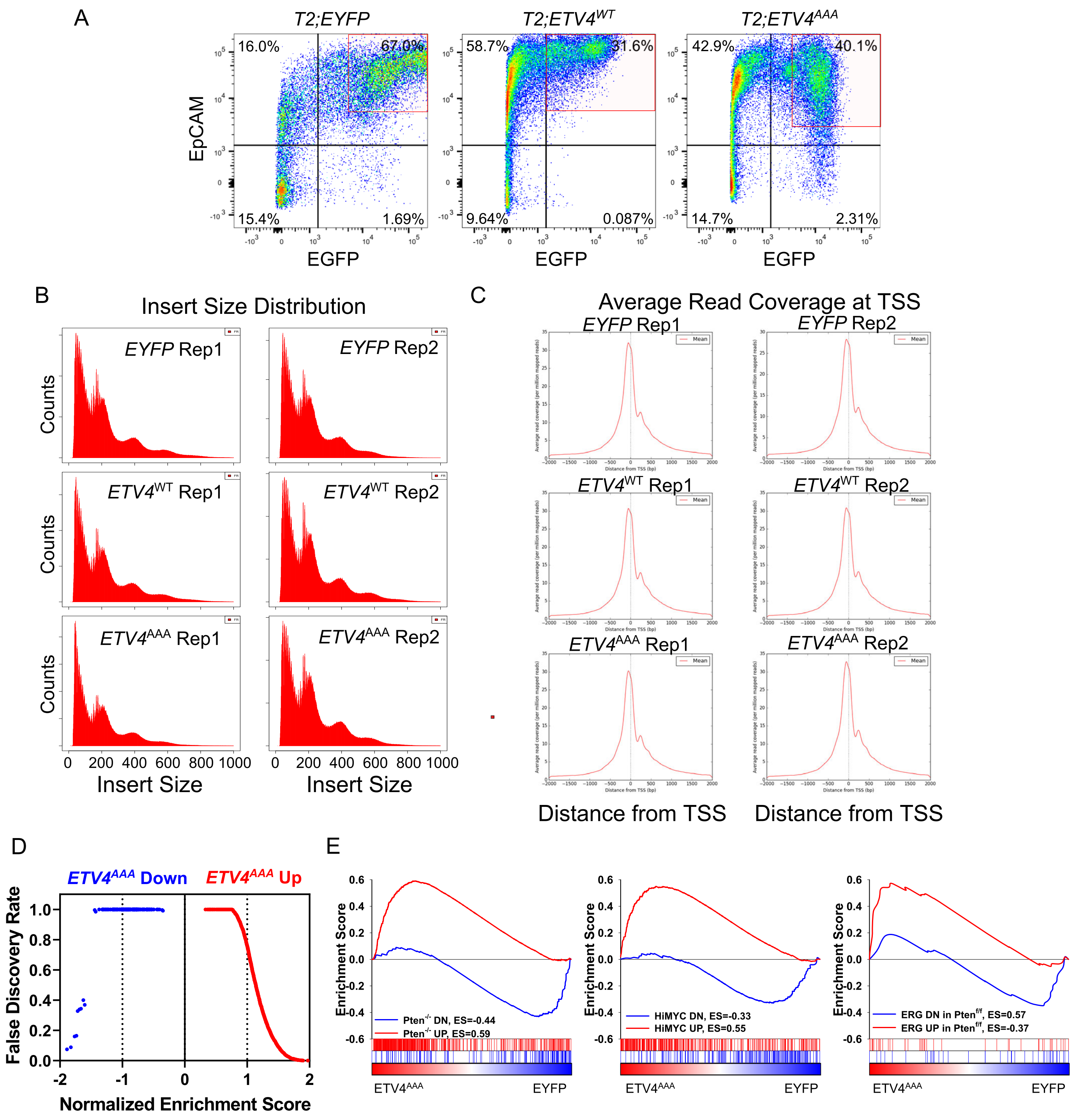
A
T2;EYFP
T2;ETV4WT
T2;ETV4AAA
EpCAM
16.0%
67.0%
15.4%
1.69%
58.7%
31.6%
9.64%
0.087%
42.9%
40.1%
14.7%
2.31%
EGFP
B
Insert Size Distribution
EYFP Rep1
EYFP Rep2
ETV4WT Rep1
ETV4WT Rep2
ETV4AAA Rep1
ETV4AAA Rep2
Counts
0 200 400 600 800 1000
Insert Size
C
Average Read Coverage at TSS
EYFP Rep1
EYFP Rep2
ETV4WT Rep1
ETV4WT Rep2
ETV4AAA Rep1
ETV4AAA Rep2
Distance from TSS
D
ETV4AAA Down
ETV4AAA Up
False Discovery Rate
Normalized Enrichment Score
E
Enrichment Score
Pten-/- DN, ES=-0.44
Pten-/- UP, ES=0.59
HiMYC DN, ES=-0.33
HiMYC UP, ES=0.55
ERG DN in Ptenf/f, ES=0.57
ERG UP in Ptenf/f, ES=-0.37
ETV4AAA
EYFP

Figure S4

Fig. S4. Insert size distribution of open-chromatin and nucleosome peaks enriched around known promoters are expected

(A) EpCAM-positive and EGFP-positive prostate cells in *T2; EYFP*, *T2; ETV4*^{WT} and *T2; ETV4*^{AAA} mice 1 week after tamoxifen treatment are analyzed by FACS sorting. The mean cell ratios of 3 replicates are labeled. (B) ATAC sequencing reads from FACS sorted EYFP, ETV4^{WT} and ETV4^{AAA} cells exhibited expected insert size distribution of open-chromatin. (C) ATAC sequencing reads from FACS sorted EYFP, ETV4^{WT} and ETV4^{AAA} cells exhibited expected nucleosome peaks enriched around known promoters. (D) Normalized enrichment scores are shown from Gene Set Enrichment Analysis with ETV4^{AAA} expressing cells compared with EYFP expressing cells using ~3,000 curated genes sets and custom prostate cancer gene sets. (E) The up and down regulated genes in ETV4^{AAA} expressing cells compared with EYFP expressing cells are enriched in gene sets up-regulated in Pten$^{-/-}$, HiMYC and ERG models.

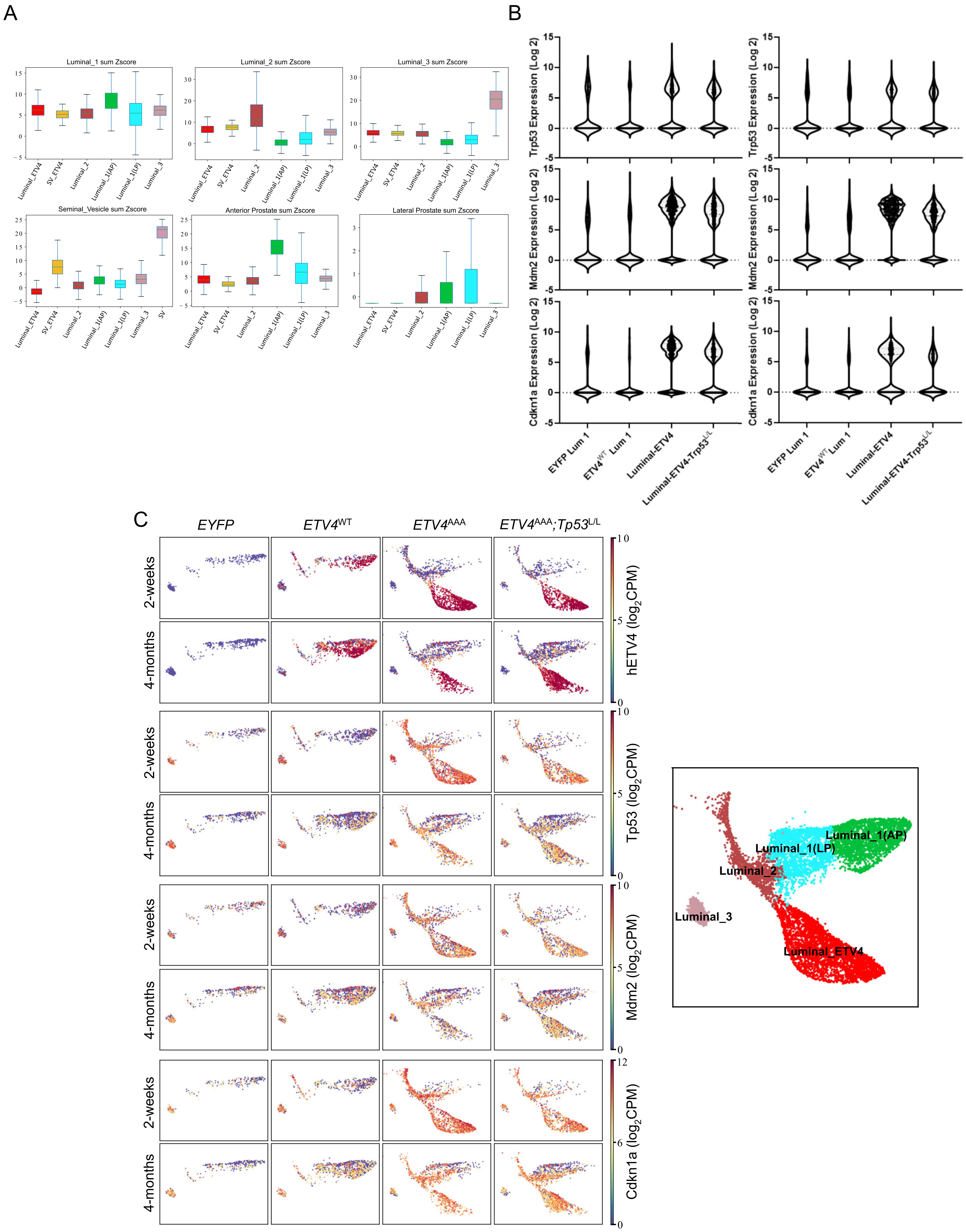
A
Luminal_1 sum Zscore
Luminal_2 sum Zscore
Luminal_3 sum Zscore
Seminal_Vesicle sum Zscore
Anterior Prostate sum Zscore
Lateral Prostate sum Zscore
Luminal_ETV4
SV_ETV4
Luminal_2
Luminal_1(AP)
Luminal_1(LP)
Luminal_3
SV
B
Trp53 Expression (Log 2)
Mdm2 Expression (Log 2)
Cdkn1a Expression (Log 2)
EYFP Lum 1
ETV4WT Lum 1
Luminal-ETV4
Luminal-ETV4-Trp53L/L
C
EYFP
ETV4WT
ETV4AAA
ETV4AAA;Tp53L/L
2-weeks
4-months
hETV4 (log2CPM)
Tp53 (log2CPM)
Mdm2 (log2CPM)
Cdkn1a (log2CPM)
Luminal_1(AP)
Luminal_1(LP)
Luminal_2
Luminal_3
Luminal_ETV4

Figure S5

Fig. S5. The expression of Tp53 and its downstream genes in ETV4^{AAA};Tp53$^{L/L}$ mice

(A) Sum of Z score of marker genes of luminal clusters. (B) Violin plot shows the mRNA expression of Tp53 and its downstream genes, Mdm2 and Cdkn1a, in Luminal_ETV4 and Luminal_1(AP+LP) clusters in *T2; EYFP*, *T2; ETV4^{WT}*, *T2; ETV4^{AAA}* and *T2; ETV4^{AAA};Tp53*$^{L/L}$ mice. (C) Scatter plot shows the expression of ETV4, Tp53, Mdm2 and Cdkn1a in luminal clusters in *T2; EYFP*, *T2; ETV4^{WT}*, *T2; ETV4^{AAA}* and *T2; ETV4^{AAA};Tp53*$^{L/L}$ mice.

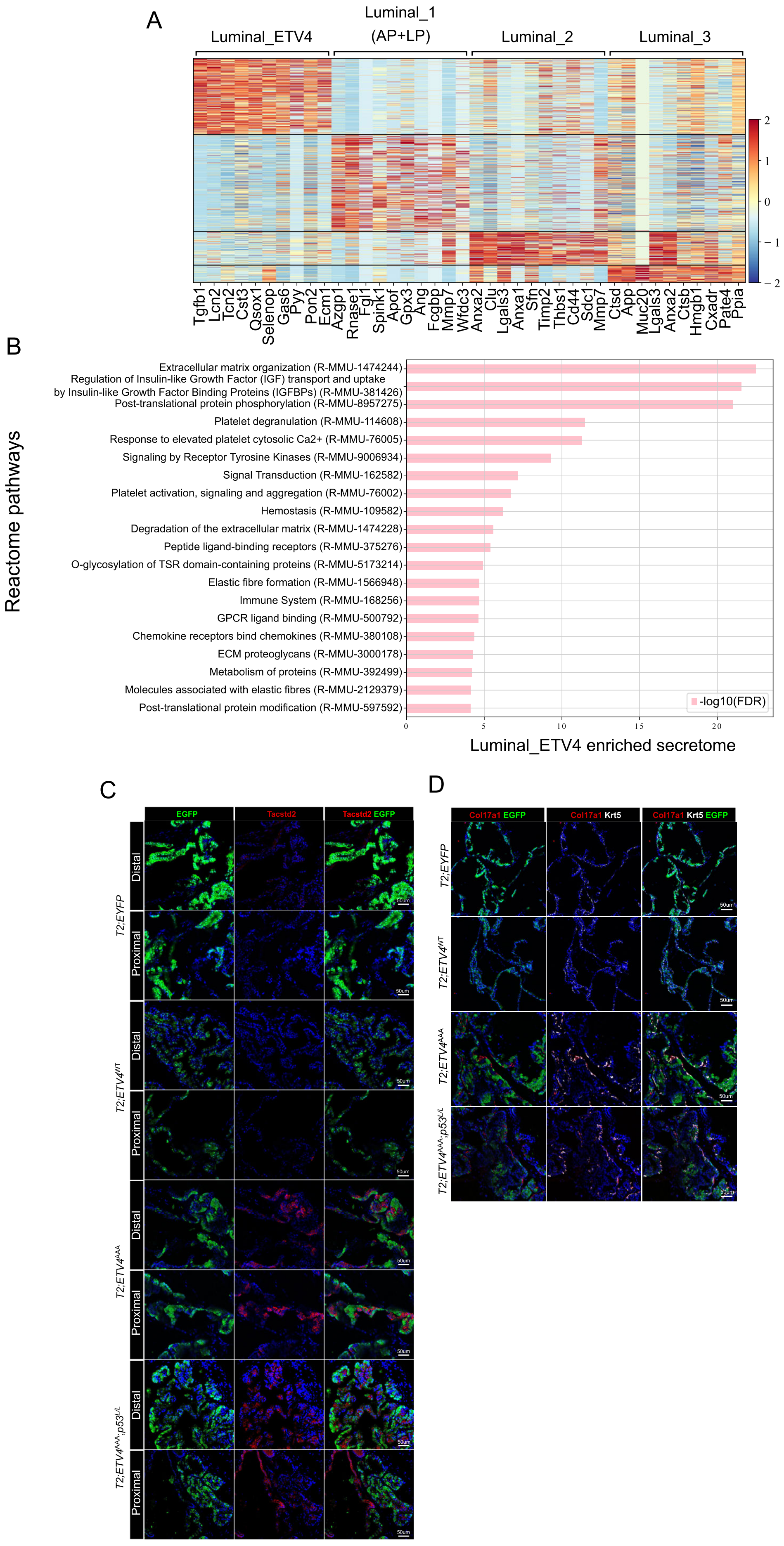
A
Luminal_ETV4
Luminal_1 (AP+LP)
Luminal_2
Luminal_3
Tgfb1
Lcn2
Tcn2
Cst3
Qsox1
Selenop
Gas6
Pyy
Pon2
Ecm1
Azgp1
Rnase1
Fgl1
Spink1
Apof
Gpx3
Ang
Fcgbp
Mmp7
Wfdc3
Anxa2
Clu
Lgals3
Anxa1
Sfn
Timp2
Thbs1
Cd44
Sdc1
Mmp7
Ctsd
App
Muc20
Lgals3
Anxa2
Ctsb
Hmgb1
Cxadr
Pate4
Ppia
B
Reactome pathways
Extracellular matrix organization (R-MMU-1474244)
Regulation of Insulin-like Growth Factor (IGF) transport and uptake by Insulin-like Growth Factor Binding Proteins (IGFBPs) (R-MMU-381426)
Post-translational protein phosphorylation (R-MMU-8957275)
Platelet degranulation (R-MMU-114608)
Response to elevated platelet cytosolic Ca2+ (R-MMU-76005)
Signaling by Receptor Tyrosine Kinases (R-MMU-9006934)
Signal Transduction (R-MMU-162582)
Platelet activation, signaling and aggregation (R-MMU-76002)
Hemostasis (R-MMU-109582)
Degradation of the extracellular matrix (R-MMU-1474228)
Peptide ligand-binding receptors (R-MMU-375276)
O-glycosylation of TSR domain-containing proteins (R-MMU-5173214)
Elastic fibre formation (R-MMU-1566948)
Immune System (R-MMU-168256)
GPCR ligand binding (R-MMU-500792)
Chemokine receptors bind chemokines (R-MMU-380108)
ECM proteoglycans (R-MMU-3000178)
Metabolism of proteins (R-MMU-392499)
Molecules associated with elastic fibres (R-MMU-2129379)
Post-translational protein modification (R-MMU-597592)
-log10(FDR)
Luminal_ETV4 enriched secretome
C
EGFP
Tacstd2
Tacstd2 EGFP
Distal
Proximal
T2;EYFP
T2;ETV4WT
T2;ETV4AAA
T2;ETV4AAA;p53L/L
D
Col17a1 EGFP
Col17a1 Krt5
Col17a1 Krt5 EGFP

Figure S6

Fig. S6. Secretome and paracrine effect of Luminal_ETV4 cells

(A) Heatmap shows the highly expressed secretome genes of luminal clusters. (B) Bar graph shows the enriched secretome pathways in Luminal_ETV4 cluster found by GO analysis. (C, D) The protein level of Tacstd2/Trop2 (A) and Col17a1, Krt5 (B) are shown with IF staining. Scale bar 50 um.

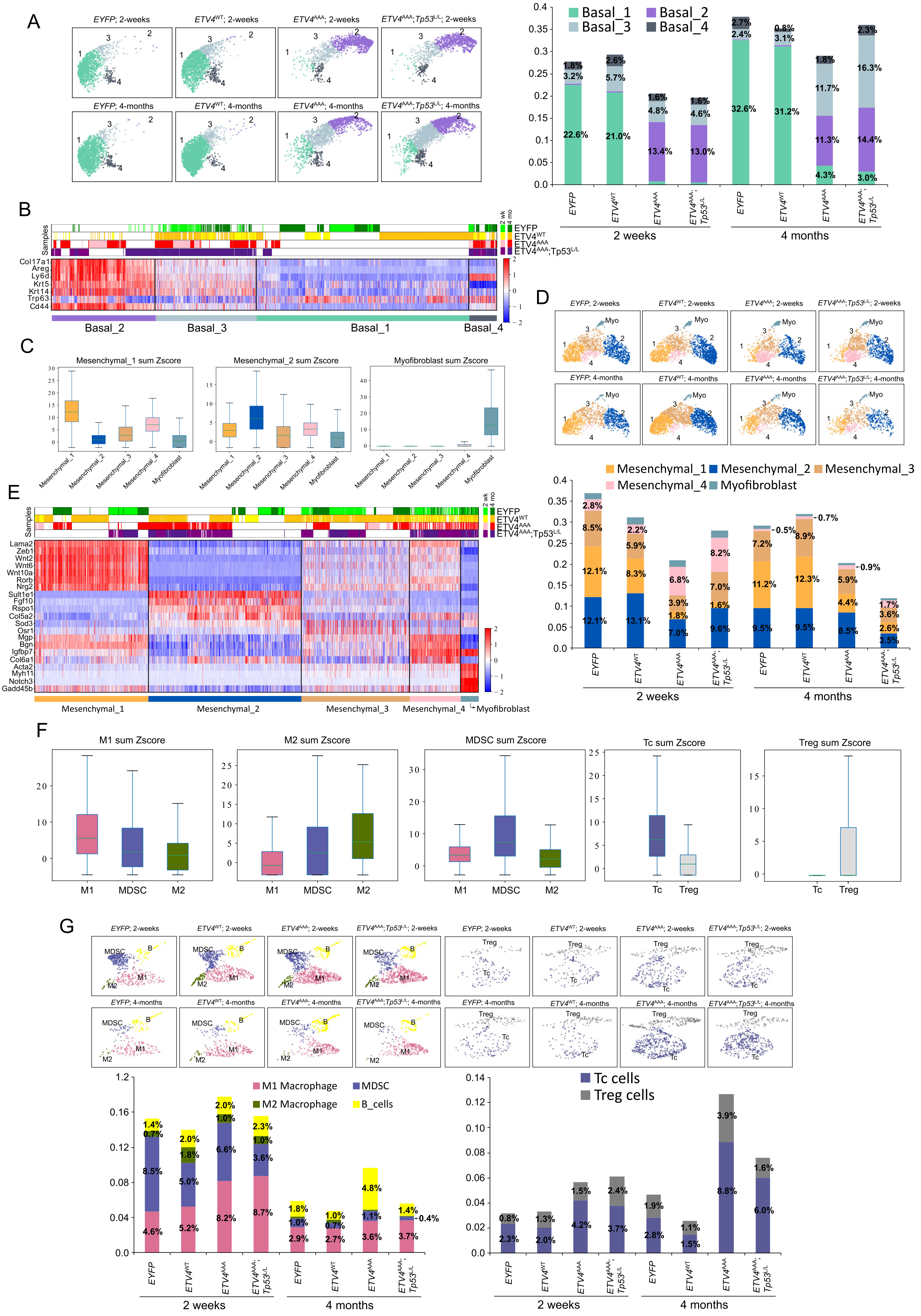
A
EYFP; 2-weeks
ETV4WT; 2-weeks
ETV4AAA; 2-weeks
ETV4AAA;Tp53L/L; 2-weeks
EYFP; 4-months
ETV4WT; 4-months
ETV4AAA; 4-months
ETV4AAA;Tp53L/L; 4-months
Basal_1
Basal_2
Basal_3
Basal_4
2 weeks
4 months
B
Samples
EYFP
ETV4WT
ETV4AAA
ETV4AAA;Tp53L/L
Col17a1
Areg
Ly6d
Krt5
Krt14
Trp63
Cd44
Basal_2
Basal_3
Basal_1
Basal_4
C
Mesenchymal_1 sum Zscore
Mesenchymal_2 sum Zscore
Myofibroblast sum Zscore
Mesenchymal_1
Mesenchymal_2
Mesenchymal_3
Mesenchymal_4
Myofibroblast
D
Myo
Mesenchymal_1
Mesenchymal_2
Mesenchymal_3
Mesenchymal_4
Myofibroblast
2 weeks
4 months
E
Samples
Lama2
Zeb1
Wnt2
Wnt6
Wnt10a
Rorb
Nrg2
Sult1e1
Fgf10
Rspo1
Col5a2
Sod3
Osr1
Mgp
Bgn
Igfbp7
Col6a1
Acta2
Myh11
Notch3
Gadd45b
Mesenchymal_1
Mesenchymal_2
Mesenchymal_3
Mesenchymal_4
Myofibroblast
F
M1 sum Zscore
M2 sum Zscore
MDSC sum Zscore
Tc sum Zscore
Treg sum Zscore
M1
MDSC
M2
Tc
Treg
G
MDSC
B
M1
M2
Treg
Tc
M1 Macrophage
MDSC
M2 Macrophage
B_cells
Tc cells
Treg cells
2 weeks
4 months

Figure S7

Fig. S7. A single-cell atlas of the mPin induced by ETV4^{AAA}

(A) Basal subsets and are shown separately for each sample and cell numbers are quantified. (B) Heatmap shows the highly differentially expressed genes for each Basal clusters. The gene expression matrix was normalized to CPM and $\log_2$(CPM+1), gene imputation was performed. (C) Sum of Z score of marker genes of stroma clusters. (D) Stroma subsets and are shown separately for each sample and cell numbers are quantified. (E) Heatmap shows the highly differentially expressed genes for each stroma clusters. The gene expression matrix was normalized to CPM and log2(CPM+1), gene imputation was performed. (F) Sum of Z score of marker genes of macrophage and T cell clusters. (G) Macrophage, B cell and T cell subsets and are shown separately for each sample and cell numbers are quantified.

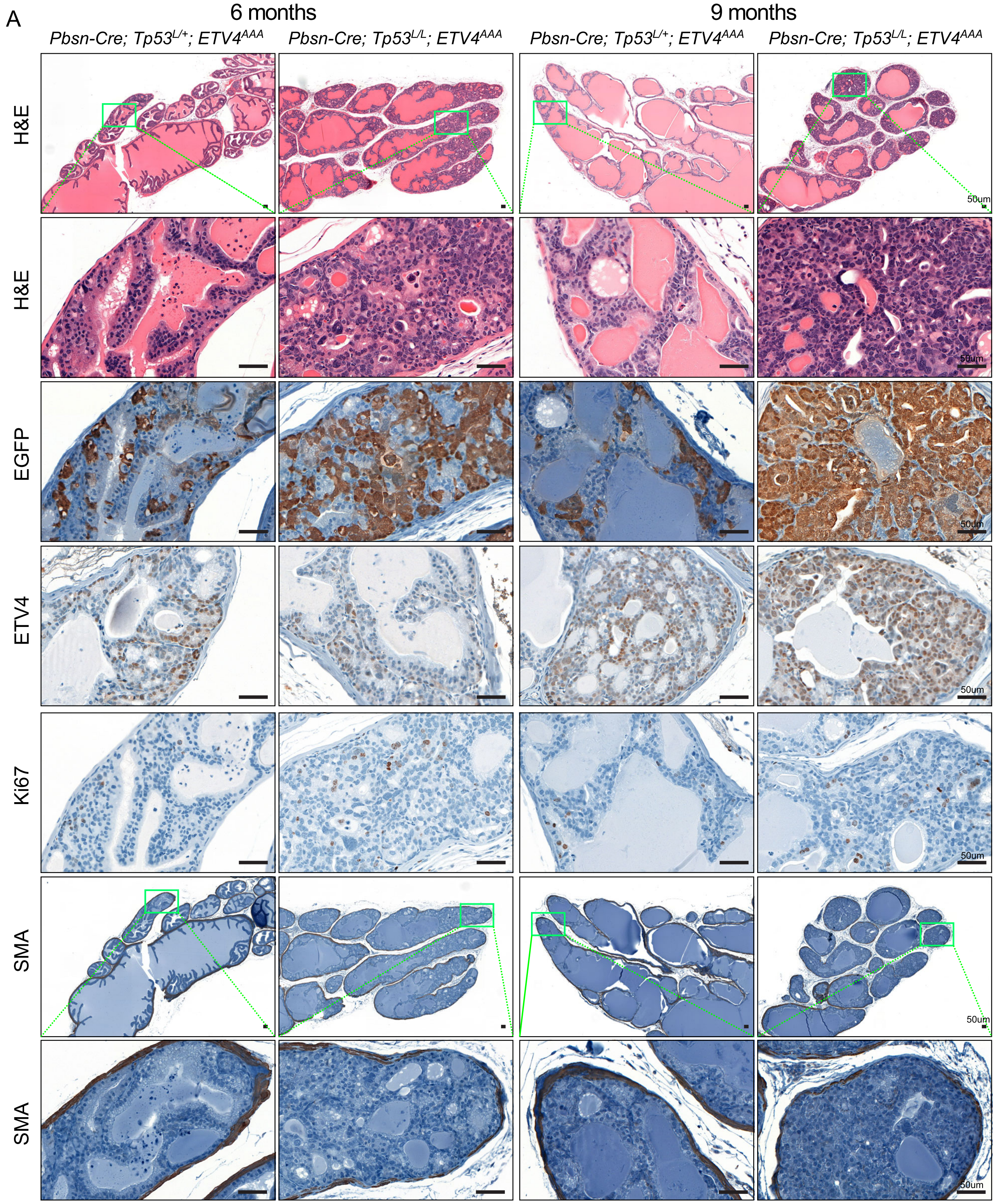
A
6 months
9 months
Pbsn-Cre; Tp53L/+; ETV4AAA
Pbsn-Cre; Tp53L/L; ETV4AAA
Pbsn-Cre; Tp53L/+; ETV4AAA
Pbsn-Cre; Tp53L/L; ETV4AAA
H&E
H&E
EGFP
ETV4
Ki67
SMA
SMA
50um

Figure S8

Fig. S8. loss Trp53 suppresses ETV4^{AAA} induced senescence and induces prostate cancer

(A) Representative H&E (low magnification and high magnification of indicated area) and IHC of GFP (antibody recognizes both EGFP and EYFP), ETV4, Ki67 and smooth muscle actin (SMA) in anterior prostate 6 months old and 9 months old *Pbsn-Cre; Tp53$^{L/+}$; ETV4^{AAA}* and *Pbsn-Cre; Tp53$^{L/L}$; ETV4^{AAA}* mice. Scale bar 50 um.

Additional files related to the supplementary materials:

Table S1. MEME-ChIP
Table S2. Bindetect_results
Table S3. Custom gene sets.
Table S4. Rank genes in clusters
Table S5. Cluster_cell_numbers
Table S6. Marker genes for Zscore
Table S7. Rank gene list Luminal_1_ETV4 vs Luminal_1(AP)
Table S8. Rank gene list ETV4AAAp53KO vs ETV4AAA 2 weeks
Table S9. Rank gene list ETV4AAAp53KO vs ETV4AAA 4 months
